# Cancer Immune Responsiveness and MHC Class I Antigen Presentation: Mechanisms of Immune Escape and Immunotherapy Resistance in Gastrointestinal Cancers

**DOI:** 10.3390/cells15171513

**Published:** 2026-08-22

**Authors:** Fabio Grizzi, Maurizio Chiriva-Internati, Mohamed A. A. A. Hegazi, Federica Rubbino, Fabio Pasqualini, Marco Spadaccini, Marta Andreozzi, Miriana Mercurio, Federico Cassano, Maria Terrin, Cesare Hassan, Robert S. Bresalier, Alessandro Repici, Silvia Carrara

**Affiliations:** 1 Department of Immunology and Inflammation, IRCCS Humanitas Research Hospital, 20089 Milan, Italy; lunamohamed75@gmail.com (M.A.A.A.H.); fabio.pasqualini@hunimed.eu (F.P.); 2 Department of Biomedical Sciences, Humanitas University, Pieve Emanuele, 20072 Milan, Italy; cesare.hassan@hunimed.eu (C.H.); alessandro.repici@hunimed.eu (A.R.); silvia.carrara@humanitas.it (S.C.); 3 Departments of Gastroenterology, Hepatology & Nutrition, Division of Internal Medicine, The University of Texas MD Anderson Cancer Center, Houston, TX 77030, USA; rbresali@mdanderson.org; 4 Laboratory of Macrophage Dynamics, IRCCS Humanitas Research Hospital, Via Manzoni 56, 20089 Rozzano, Italy; federica.rubbino@humanitasresearch.it; 5 Division of Gastroenterology and Digestive Endoscopy, Department of Gastroenterology, IRCCS Humanitas Research Hospital, Via Manzoni 56, 20089 Rozzano, Italy; marco.spadaccini@humanitas.it (M.S.); marta.andreozzi@humanitas.it (M.A.); miriana.mercurio@humanitas.it (M.M.); federico.cassano@humanitas.it (F.C.);

**Keywords:** HLA class I, APM, cancer, immune system, tumour escape, gastrointestinal neoplasms, neoantigen, IFN-γ signalling, epigenetic regulation

## Abstract

**Highlights:**

**What are the main findings?**

- Reduced or absent HLA class I expression occurs in 30–76% of gastrointestinal tumours, depending on tumour site and molecular subtype, and results from distinct defects affecting four functional levels.
- Across cancers, our analysis indicates that APM dysregulation is largely established early in tumour development rather than acquired during disease progression. Moreover, bulk transcriptomic profiles may not accurately reflect tumour-cell protein expression, as demonstrated in oesophageal carcinoma.

**What is the implication of the main finding?**

- Because only some defects are reversible, patients should be stratified according to the underlying mechanism of HLA class I loss rather than simply its presence or absence: reversible defects may be addressed by restoring antigen presentation, whereas irreversible defects require strategies that bypass it.
- This approach requires cell-resolved, quantitative assessment of the APM, prospectively incorporated into immunotherapy trials as a stratification factor for patients with gastrointestinal cancer.

**Abstract:**

The Antigen Processing and Presentation Machinery (APM) is essential for immune surveillance by enabling the presentation of antigenic peptides to T lymphocytes and facilitating the elimination of infected or transformed cells. In cancer, the integrity of this process influences cancer immune responsiveness (CIR), defined as a tumour’s capacity to be recognised by the immune system and respond to immunotherapy. Tumours with intact antigen presentation pathways are more likely to generate effective antitumour responses, whereas APM defects promote immune escape and therapeutic resistance. Cancer cells frequently evade immune detection through altered antigen processing or reduced expression of major histocompatibility complex (MHC) class I molecules, limiting tumour antigen presentation to cytotoxic T lymphocytes. These alterations are increasingly recognised as determinants of response to immune checkpoint inhibitors and potential predictive biomarkers. APM defects may be reversible or irreversible. Interferon-mediated signalling can restore MHC class I expression and T-cell cytotoxicity in some tumours, whereas permanent genomic alterations affecting human leukocyte antigen (HLA) class I genes, β2-microglobulin (β2-m), or interferon-γ (IFN-γ) pathway components can severely impair antigen presentation. Emerging evidence highlights four mechanistic levels of APM perturbation: peptide generation, peptide loading, MHC class I integrity, and epigenetic regulation. Each contributes to distinct patterns of immune evasion. This review examines how MHC class I alterations influence CIR and contribute to immune evasion and immunotherapy resistance in gastrointestinal malignancies, while discussing therapeutic strategies to restore or bypass APM deficiencies.

## 1. Introduction

Immune surveillance of malignant cells requires the presentation of tumour-derived antigens through major histocompatibility complex (MHC) class I molecules and the consequent activation of cytotoxic CD8^+^ T cells [1,2,3,4]. This pathway depends critically on the proper functioning of the Antigen Processing and Presentation Machinery (APM), a coordinated network of cellular components and signalling cascades that ensure efficient antigen processing and display [5,6,7,8,9,10,11]. In gastrointestinal (GI) cancers, defects in APM components, ranging from partial impairment to complete loss, are frequently observed and are now recognised as a major strategy of tumour immune evasion. These alterations arise through both reversible mechanisms, such as epigenetic silencing and dysregulated signalling pathways, and irreversible events, including somatic mutations, gene deletions, and structural genomic rearrangements [12,13]. With immunotherapy, particularly immune checkpoint inhibitors (ICIs), now central to cancer treatment, there is an urgent need to clarify how dysfunction of the APM restricts therapeutic response and how these defects might be restored or circumvented.

Notably, recent pan-cancer analyses have revealed that HLA-I loss of heterozygosity (LOH) is frequent across diverse tumour types [14], and β2-microglobulin (β2-m) mutations represent a prevalent mechanism of acquired resistance to both CTLA-4- and PD-1-targeted therapies. Functional classification of APM perturbations into four mechanistic layers, peptide generation, peptide loading, MHC class I integrity, and epigenetic regulation, provides a framework to identify alterations that are most clinically actionable [15].

In this context, we present a narrative review on APM disruption in GI cancers. We examine the molecular basis of antigen presentation, review approaches for assessing APM status, and discuss how APM loss contributes to immune escape and resistance to immunotherapy. We further integrate insights from recent advances in neoantigen vaccine design and immunopeptidomics that are reshaping the understanding of how antigen presentation defects influence therapeutic outcomes.

## 2. Molecular Framework of Antigen Presentation and the APM

**Figure 1:**
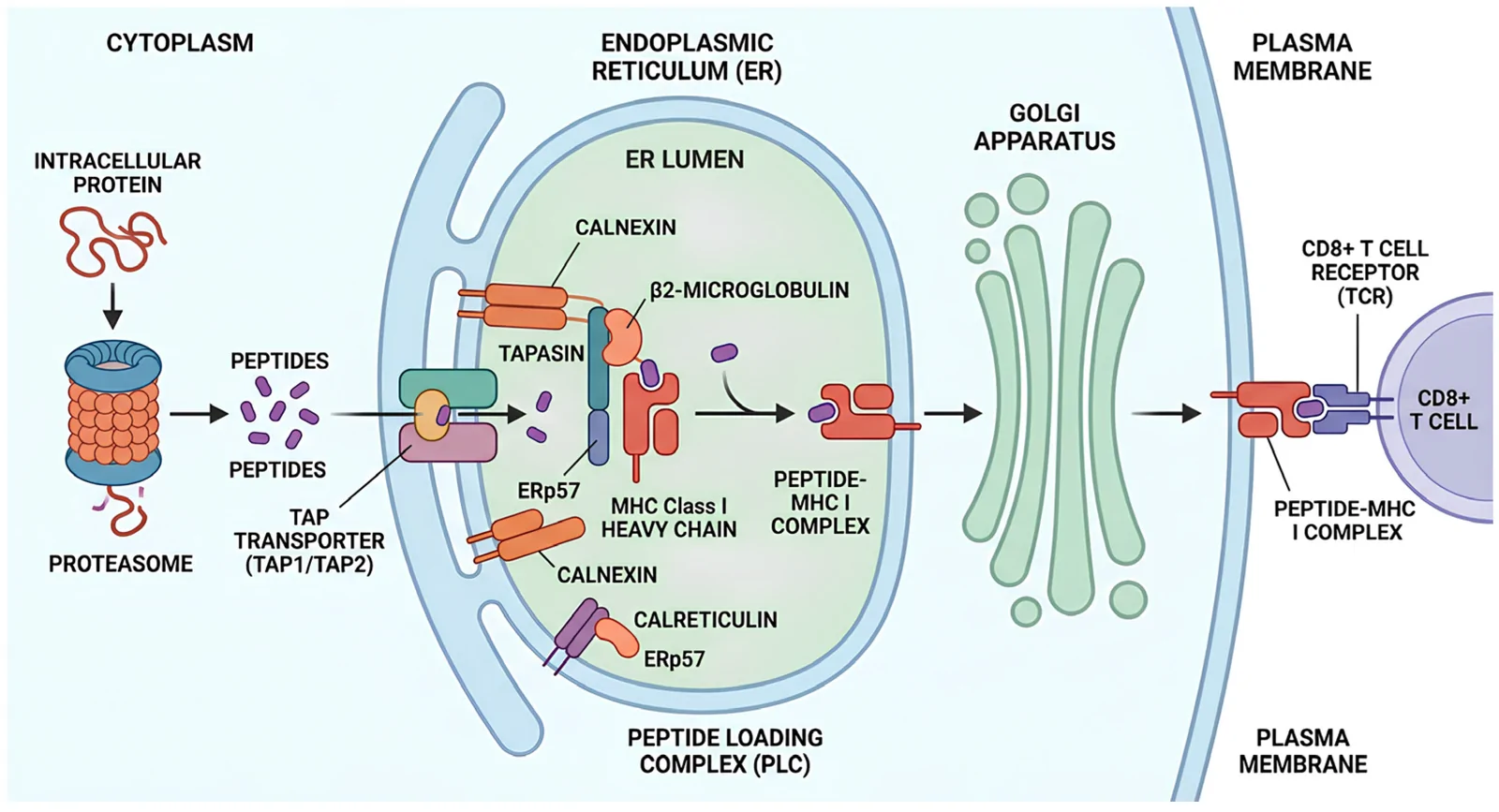
Antigen processing and presentation through the MHC class I pathway. Intracellular proteins are degraded by the proteasome into peptide fragments within the cytoplasm. These peptides are transported into the ER lumen through the TAP1/TAP2 transporter complex. Within the ER, newly synthesised MHC-I heavy chains associate with β2-microglobulin and components of the peptide-loading complex (PLC), including calnexin, calreticulin, ERp57, and tapasin, to facilitate proper folding and peptide loading. The resulting peptide–MHC-I complex is then transported through the Golgi apparatus to the plasma membrane, where it is presented to CD8^+^ T cells via the TCR, thereby enabling immune surveillance and cytotoxic T-cell activation.

The MHC class I antigen presentation pathway is initiated by the proteasomal degradation of intracellular proteins (Figure 1). The resulting peptides are subsequently transported into the endoplasmic reticulum (ER) via the TAP1 and TAP2 transporter complex [16]. Within the ER, these peptides are loaded onto the MHC class I heavy chain (MHC-I-HC), which is stabilised by β2-m. Proper folding and efficient peptide loading are ensured by a set of dedicated chaperone proteins, including calnexin [17], calreticulin, tapasin, and ERp57. Following assembly, the peptide–MHC class I complex is transported to the cell surface, where it is surveyed by the T-cell receptor (TCR) on CD8^+^ T lymphocytes. A central component of this process is tapasin, which mediates peptide editing and thereby shapes the quality and repertoire of peptides presented to cytotoxic T lymphocytes (CTLs) [14,18,19].

### Mechanistic Levels of APM Perturbation

Recent functional analyses have organised APM perturbations into four interconnected mechanistic levels, each with distinct consequences for antigen presentation and immune evasion [15]:

(a) Peptide generation: Disruption at the level of proteasomal degradation and immunoproteasome function. The immunoproteasome, composed of the interferon-γ (IFN-γ) inducible subunits LMP2 (PSMB9/β1i), LMP10 (PSMB10/β2i), and LMP7 (PSMB8/β5i), generates peptides better suited for MHC class I presentation. Downregulation of these subunits, frequently observed across GI cancers, directly reduces the repertoire of peptides available for CD8^+^ T-cell recognition. Similarly, deficiencies in ERAP1 and ERAP2, the ER aminopeptidases that perform N-terminal peptide trimming before loading, compromise peptide quality and MHC-I stability [15].

(b) Peptide loading: Dysfunction of the peptide-loading complex (PLC), including TAP1/TAP2, tapasin, calreticulin, and ERp57. TAP deficiency impairs transport of cytosolic peptides into the ER lumen, while tapasin loss reduces peptide editing efficiency and diminishes stable pMHC-I surface expression. Reduced calreticulin expression in both murine and human models has been linked to a 50–80% reduction in the surface expression of HLA class I–peptide complexes [14].

(c) MHC class I integrity: Structural disruptions including mutations or deletions in HLA-I heavy chain genes or β2-m. Loss of β2-m is a well-established mechanism of acquired ICI resistance, observed across multiple tumour types. HLA-I LOH is detected in 17–45% of diverse tumour types and is specifically enriched for alleles presenting driver neoantigens [14]. Alternative splicing of HLA genes and secretion of soluble HLA (sHLA) molecules further compromise surface antigen presentation.

(d) Epigenetic regulation: Transcriptional silencing of APM components through DNA methylation, histone deacetylation, and polycomb repressive complex 2 (PRC2/EZH2)-mediated chromatin compaction. Epigenetic mechanisms account for the majority of APM defects across cancer types [11] and are important because they are potentially reversible with targeted therapies, unlike structural genomic alterations.

Disruption of these pathways compromises immune recognition and facilitates tumour immune evasion. Genetic, epigenetic, and post-transcriptional mechanisms may act in combination, which complicates both diagnosis and therapeutic intervention [12,13].

## 3. Tissue-Based Analysis of HLA Class I Antigen Processing Pathways

A variety of methods have been developed to assess the expression of HLA class I and APM components in tumour tissue. RNA-based techniques, such as RNA sequencing (RNA-seq) and NanoString, allow the detection of gene expression at the mRNA level, even from small tissue samples. However, these techniques analyse nucleic acids from heterogeneous cell populations, comprising tumour cells, stromal elements, and immune infiltrates, potentially masking the true expression levels of HLA-I and APM within malignant cells. Moreover, RNA-level data may not accurately reflect protein abundance due to post-transcriptional regulation by microRNAs and RNA-binding proteins. To assess protein expression directly, immunohistochemistry (IHC) and multiplex fluorescence (MP-FL) are widely used [20].

Emerging integrated approaches, combining immunopeptidomics with proteogenomics, offer superior resolution in characterising the antigenic landscape. Mass spectrometry-based immunopeptidomics enables unbiased identification of naturally presented HLA-bound peptides from tumour and benign tissues, directly capturing the functional output of the APM [14]. Combined with genomic and transcriptomic analyses, this approach also enables the characterisation of allele-specific loss of expression, alternative splicing of HLA genes, and soluble HLA secretion [14].

MP-FL and IHC are advanced antibody-based technologies that enable the simultaneous detection of multiple biomarkers within a single tissue section while preserving tissue architecture and spatial information. Compared with single-marker IHC, these approaches are tissue-sparing and enable the detection of co-localised markers, facilitating the characterisation of immune cell phenotypes and their spatial relationships within the tumour microenvironment (TME). However, the complexity of multiplex analyses requires rigorous standardisation and reporting practices to ensure reproducibility, validation, and comparability across studies. To address these challenges, the Society for Immunotherapy of Cancer (SITC) developed the Standards for Reporting of Multiplex Immunohistochemistry/Immunofluorescence Assays (STORMI), a consensus checklist aimed at harmonising the reporting of MP-FL/IHC studies [21]. The checklist addresses study scope and staining platform, assay validation and quality control, panel design and antibody selection, image acquisition, and segmentation and phenotyping strategies, and should be applied whenever multiplex assays are used to quantify HLA class I and APM expression [22,23].

MP-FL enables the identification of cell phenotypes and the quantification of their spatial relationships within the TME, potentially providing insights into clinical response and resistance to immunotherapy [24,25,26,27]. IHC enables visualisation of specific APM components within tissue sections using antibodies and is particularly informative when expression is uniform across tumour cells. However, interpretation can be complicated by weak or heterogeneous staining. Scoring conventions differ across studies: tumours are generally classified as positive, heterogeneous, weak, or negative according to the proportion of stained cells and staining intensity [28,29]. The XII International Histocompatibility Workshop criteria define positivity as staining in more than 75% of cells, heterogeneity as staining in 25–75% of cells, and negativity as staining in fewer than 25% of cells [12]. Stromal and tumour-infiltrating immune cells can serve as internal positive controls. Inter-patient, intratumoral, and inter-lesional heterogeneity all contribute to the variability in APM expression observed in clinical samples. MP-FL/IHC examination confirmed the heterogeneity of HLA-I and APM components, revealing the coexistence of both HLA-I/APM-positive and -negative tumour cells within the same tumour tissue samples. This finding has driven the development of new computer-aided image analysis algorithms designed to reduce observer subjectivity.

## 4. APM Alterations in Gastrointestinal Malignancies

### 4.1. Oesophageal Cancer

Oesophageal squamous cell carcinoma (ESCC) is among the most lethal forms of cancer. According to GLOBOCAN 2020, oesophageal cancer ranks seventh in incidence and sixth in mortality rates among cancers worldwide [30]. Sheyhidin et al. [31] found that although general methylation levels of genes like HLA-B, TAP2, tapasin, and ERp57 did not differ significantly between ESCC and normal tissues, specific CpG sites showed distinct methylation patterns, indicating site-specific epigenetic changes in ESCC. Effective T-cell-mediated tumour clearance requires stable expression of HLA-I complexes, which are dependent on multiple APM components for their assembly and surface presentation [32]. Tanaka et al. [33] assessed 11 ESCC cell lines using Western blot analysis and identified variable expression patterns for several key APM proteins, including HLA-HC, β2-m, TAP1, TAP2, LMP7, and tapasin. Immunohistochemical analysis of 95 tumour samples revealed that decreased HLA-HC and increased TAP1 expression were independently associated with poor prognosis, particularly in advanced stages (p-stage III/IV) [33]. In another study analysing 143 ESCC cases from Shandong Province, China, reduced expression of HLA-I and other APM molecules, such as TAP1, CANX, LMP7, ERp57, tapasin, and ERAP1, was frequently observed [34]. These deficiencies correlated with tumour grade, invasion depth, lymph node metastasis, and high-risk HPV16 infection, suggesting a role in tumour progression and immune escape [35]. Additionally, Zhang et al. [36] reported that increased HLA-F and decreased HLA-I expression were independent negative prognostic indicators. This was further validated by multivariate analysis, which confirmed that low HLA-I expression significantly correlated with reduced survival, particularly in metastatic tumours [37]. Radiotherapy not only inhibits tumour progression in ESCC but also exerts important immunomodulatory effects on the TME. Shao et al. have shown that elevated peripheral blood β2-m levels after radiotherapy were significantly associated with improved disease-free survival, while radiation exposure increased HLA-I expression in ESCC cells at both mRNA and protein levels [38]. Transcriptomic analyses identified cytokine–cytokine receptor interaction pathways as highly enriched, with IFN-γ emerging as a central regulator of HLA-I expression; additionally, radiation enhanced IFN-γ secretion and altered dendritic cell and T-cell distribution in co-culture systems, suggesting that radiotherapy promotes antitumour immunity through IFN-γ-mediated immune activation.

The reduced protein-level expression of HLA class I and APM components repeatedly documented in ESCC by immunohistochemistry and immunoblotting contrasts with the transcriptomic profile obtained from The Cancer Genome Atlas (Section 5), in which most APM transcripts are significantly increased in oesophageal tumours relative to normal tissue, a pattern superficially resembling that observed in hepatocellular carcinoma (HCC). This apparent discordance can be explained by several considerations. First, bulk transcriptomic data are derived from whole-tissue lysates, in which tumour-infiltrating lymphocytes, stromal cells, and endothelial cells, all of which express HLA class I and APM genes at relatively high levels, contribute substantially to the measured signal, whereas immunohistochemistry enables assessment of expression at the cellular level and can distinguish malignant cells from the surrounding microenvironment. Given that ESCC is typically a densely inflamed tumour, the bulk transcriptional profile may, therefore, substantially reflect the contribution of the immune and stromal compartments. Second, the normal reference tissue in the oesophageal cohort is squamous mucosa, which has relatively low-baseline APM expression. An IFN-γ-rich microenvironment can consequently produce a net transcriptional increase even when tumour cells themselves remain APM-deficient. Third, transcript abundance is an imperfect surrogate for the abundance of surface peptide–HLA complexes because post-transcriptional mechanisms (e.g., MEX3B and microRNAs), post-translational mechanisms (e.g., aberrant glycosylation and lysosomal degradation), and structural alterations (e.g., β2-m mutation and HLA class I loss of heterozygosity) can uncouple mRNA abundance from functional antigen presentation [11,14,39]. The situation, therefore, differs substantively from hepatocellular carcinoma, in which upregulation has also been documented at the protein level within malignant hepatocytes and is accompanied by increased CD8^+^ T-cell infiltration. This comparison is, therefore, instructive rather than contradictory: it illustrates why transcriptomic analyses of APM status should be interpreted alongside cell-resolved protein-level data.

### 4.2. Gastric Cancer

Gastric cancer is not among the top ten most common cancers in the United States, yet it remains one of the leading causes of cancer-related deaths globally. Variations in tumour biology between Eastern and Western populations further complicate the development of universally applicable standard-of-care treatments based on international clinical trials [40]. Mimura et al. demonstrated that HLA-A expression, a key element of the HLA-I complex, is primarily regulated by the mitogen-activated protein kinase (MAPK) signalling pathway in both gastric and oesophageal cancers [41]. Targeting this axis, therefore, modulates tumour immunogenicity through HLA-I presentation [41]. Importantly, recent pan-cancer data show that in patients with MSI-H gastric cancer, the prevalence of HLA-I total loss at the tumour centre reaches 45.1%, with substantial prognostic implications depending on CD8^+^ TIL infiltration and PD-L1 co-expression [14].

In another study, Hirata et al. assessed 60 stage-matched gastric cancer cases and found that microsatellite instability-high (MSI-H) tumours displayed frequent mutations in β2-m and APM components, such as TAP1, TAP2, LMPs, and tapasin, defects not present in microsatellite stable (MSS) tumours [42]. These findings support the idea that MSI-H tumours are more prone to impaired antigen presentation. Further analyses revealed complete loss of HLA-I molecules in 9.6% of gastric cancers, with selective HLA-B loss in 3.4% [43]. Additionally, HLA-I expression inversely correlated with tumour depth, and LOH at 6p21.3 was linked to reduced HLA-A levels [43,44]. The absence of HLA-DR due to CIITA silencing and consistent HLA-I loss in lymph node metastases further emphasised its prognostic value [45,46].

### 4.3. Colorectal Cancer

The mechanisms behind the loss of MHC-I expression in various tumours, including CRCs, are not yet fully understood. However, studies from 2003 identified two major causes for the complete absence of MHC surface expression in CRCs: mutations in β2-m and the downregulation of LMP7 and TAP2 proteins, both crucial for antigen processing and presentation. These defects impair T lymphocyte recognition and weaken immune responses against tumours [47].

To explore the potential link between oncogenic *ras* mutations and the downregulation of APM, a focused study analysed KRAS mutations via allele-specific restriction analysis in 10 cases of high-grade intraepithelial neoplasia, alongside primary tumours and lymph node metastases from 42 CRC patients [48]. The study also used IHC to assess APM component expression and tumour cell proliferation. Results indicated that APM deficiencies were more prevalent in tumours harbouring KRAS mutations, and their coexistence correlated with advanced disease stages, suggesting that KRAS mutations may help tumours evade immune surveillance by suppressing MHC-I antigen processing.

In CRCs exhibiting the MSI-H phenotype, a strong immune response is noted due to the accumulation of frameshift mutations in coding microsatellites (cMS), which generate novel tumour-specific frameshift peptides (FSPs). These neo-peptides trigger potent cytotoxic immune reactions. A comparative study of 20 MSI-H versus 20 MSS CRCs using a panel of monoclonal antibodies against APM components revealed that complete loss of HLA-I antigen expression occurred in 60% of MSI-H cases compared to 30% of MSS cases.

Additionally, mutations in β2-m, TAP1, and TAP2 genes were identified exclusively in MSI-H tumours (35%) and were absent in MSS tumours (*p* = 0.0002) [49]. Consistent with these findings, pan-cancer analyses of 161 MSI-H CRC patients revealed reduced HLA-A/B/C expression in 70.2% of cases and reduced β2-m expression in 42.9%, highlighting the frequent impairment of antigen presentation pathways in this molecular subtype [14]. Interestingly, reduced expression of these APM components was also associated with poor prognosis in MSS patients, suggesting that defects in antigen presentation may influence clinical outcomes beyond MSI-H disease [50]. These observations support the concept that immunoselective pressure in MSI-H CRC favours the emergence and expansion of tumour clones with impaired antigen presentation, thereby facilitating immune escape. Supporting this hypothesis, Dierssen et al. [50] demonstrated that hereditary and sporadic MSI-H tumours acquire HLA class I loss through distinct genetic pathways, indicating that divergent evolutionary mechanisms can converge on defective antigen presentation and contribute to tumour progression.

**Figure 2:**
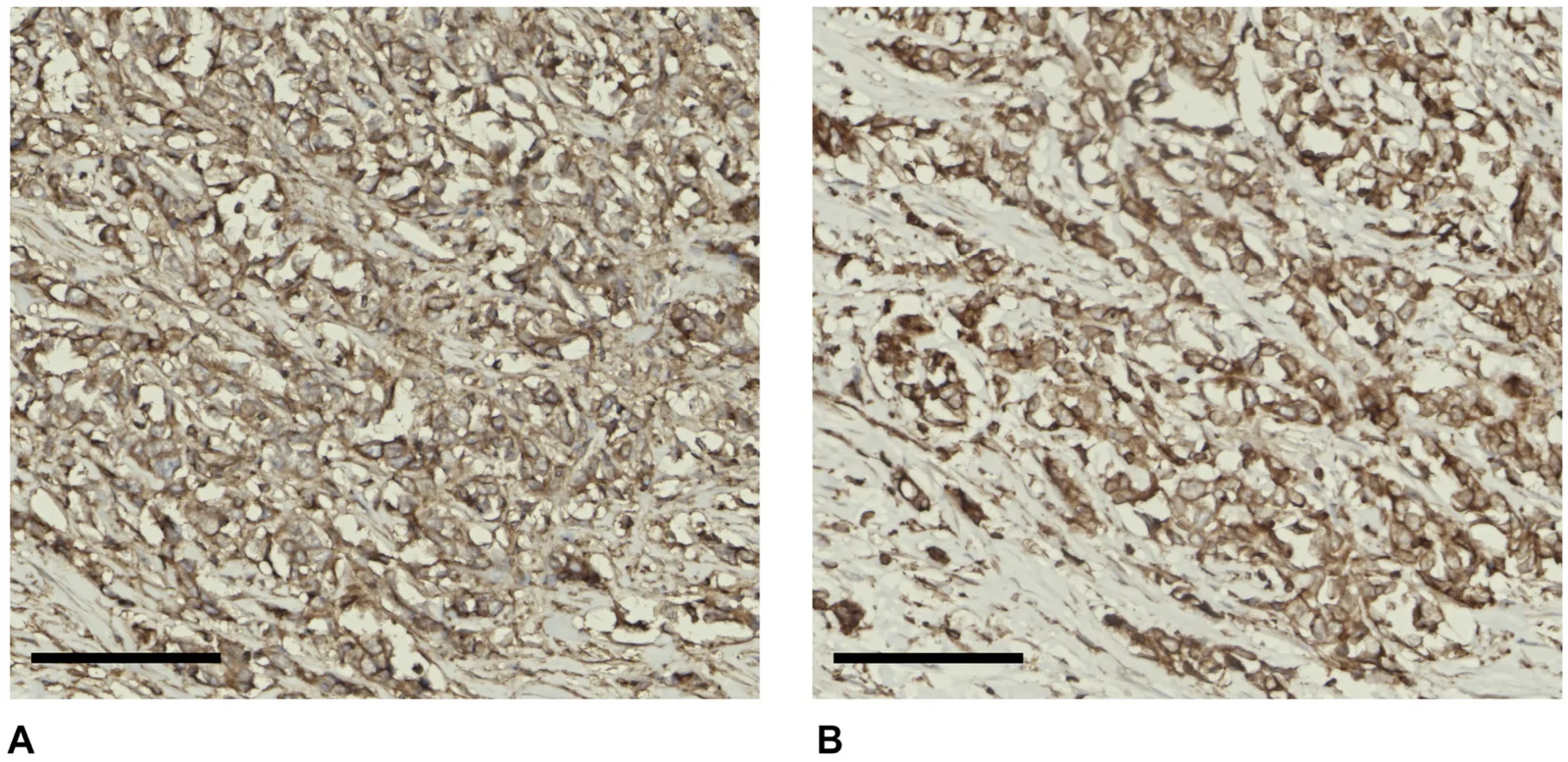
Immunohistochemical expression of HLA class I and tapasin in CRC tissue. (**A**) Representative immunohistochemical staining for HLA class I in CRC tissue, showing heterogeneous cytoplasmic and membranous expression in tumour cells. (**B**) Tapasin expression in CRC tissue, demonstrating variable cytoplasmic staining in malignant cells. Stromal cells and TILs serve as internal positive controls. Tissue specimens were obtained from the USBiomax USA tissue microarray (BCN1021). Immunohistochemical staining was performed according to standardised protocols using the mouse monoclonal anti-MHC class I antibody (Clone: EMR8-5; Cell Signaling Technology, Milan, Italy) and the rabbit monoclonal anti-tapasin antibody (Clone: E6P1H; Cell Signaling Technology, Italy). The stained slides were digitised using a Zeiss Axioscan Z1 automated slide scanner (Zeiss, Oberkochen, Germany). Scale bar: 100 µm.

Bolzacchini et al. [51] found no difference in APM signature expression between MSI-H and MSS tumours but reported elevated APM levels in CD8^+^ BRAF-mutated CRCs. This implies that integrating MSI status and CD8^+^ TILs presence may improve patient selection for ICIs. MSI-H CRCs, characterised by deficient DNA mismatch repair (dMMR), exhibit a high mutational burden, eliciting robust immune infiltration [52,53]. However, genetic alterations in APM and Wnt/β-catenin pathways may cause resistance to PD-1 blockade therapy [54,55]. The retained sensitivity of β2-m-inactivated, mismatch-repair-deficient tumours to ICIs, mediated by γδ T cells, is discussed in Section 7.2 [56]. Research on HLA-I expression and prognosis in CRC remains contradictory. Some studies associate higher HLA-I levels with improved survival through enhanced T-cell responses [57,58], while others link total HLA-I loss to favourable outcomes, possibly due to natural killer (NK) cell activity [59,60,61]. Notably, high HLA-B/C expression, rather than HLA-A, independently predicts better outcomes in colon and rectal cancers, highlighting the importance of specific HLA molecules in antitumour immunity [62]. BRAF mutations correlate with reduced HLA-I expression, potentially through MAPK pathway activation, which acts in concert with DNA methylation to silence HLA class I and NKG2D ligands [63]. Further studies have revealed that MSI-H CRCs have frequent mutations in genes encoding HLA-I components and APM [64]. Radiation upregulates MHC class I expression through interferon-mediated pathways in preclinical models, a mechanism of potential relevance to radiotherapy in rectal cancer [65,66,67]. In the same series, low HLA expression correlated with MSI and BRAF mutations but inversely with KRAS mutations, underscoring complex genetic–immune interactions [62]. Kawazu et al. [68] reported truncating mutations and allele losses in HLA-ABC genes in MSI-H CRCs, and reduced lymphocyte infiltration in some tumours without genetic defects. Loss of HLA-I expression severely limits T-cell recognition, diminishing immunotherapy efficacy [69,70]. Speetjens et al. [58] showed that HLA-I downregulation correlates with poor prognosis in rectal cancers, particularly in MSS tumours, emphasising the need to stratify MSI and MSS CRCs in prognostic assessments. Figure 2 shows representative immunohistochemical staining of HLA class I and tapasin in CRC tissue.

### 4.4. Liver Cancer

Only in a few tumours is malignancy associated with the upregulation of HLA class I antigens. Among them is HCC [71]. The frequency of HLA class I APM component upregulation and its clinical significance in HCC are not known [72]. Grizzi et al. [71] analysed 21 surgically resected primary HCC tumours alongside autologous adjacent non-malignant tissues. The study revealed that all malignant HCCs exhibited high levels of HLA class I APM components. In contrast, normal hepatocytes lacked detectable expression of these molecules, although low expression was observed in some morphologically normal hepatocytes adjacent to the tumours. Upregulation of HLA-I APM components in HCC was positively associated with the extent of CD8^+^ T-cell infiltration. This supports HCC as a candidate for T-cell-based immunotherapy [71], for which the level of HLA-I expression on tumour cells is a pivotal determinant [73]. In vitro, HLA class I and class II expression in HCC-derived cell lines can be modulated by INF-γ, sodium butyrate, and clofazimine, with marked line-to-line variability [74], and HCC cells generally retain strong HLA-I expression, which supports the rationale for CTL-based active specific immunotherapy [75]. Limited information is currently available concerning HLA-I antigen abnormalities in sarcomatoid HCC (sHCC). Lei et al. [76] investigated the growth characteristics and HLA-I antigen status of four sHCC cell lines (sHCC29, sHCC63, sHCC74, and SAR-HCV). Their analysis revealed that, unlike sHCC74 and SAR-HCV, the sHCC29 and sHCC63 cell lines lacked detectable surface expression of HLA-I antigens. This absence was accompanied by undetectable intracellular β2-m, as well as pronounced downregulation of HLA-I HC and selected components of the APM.

**Figure 3:**
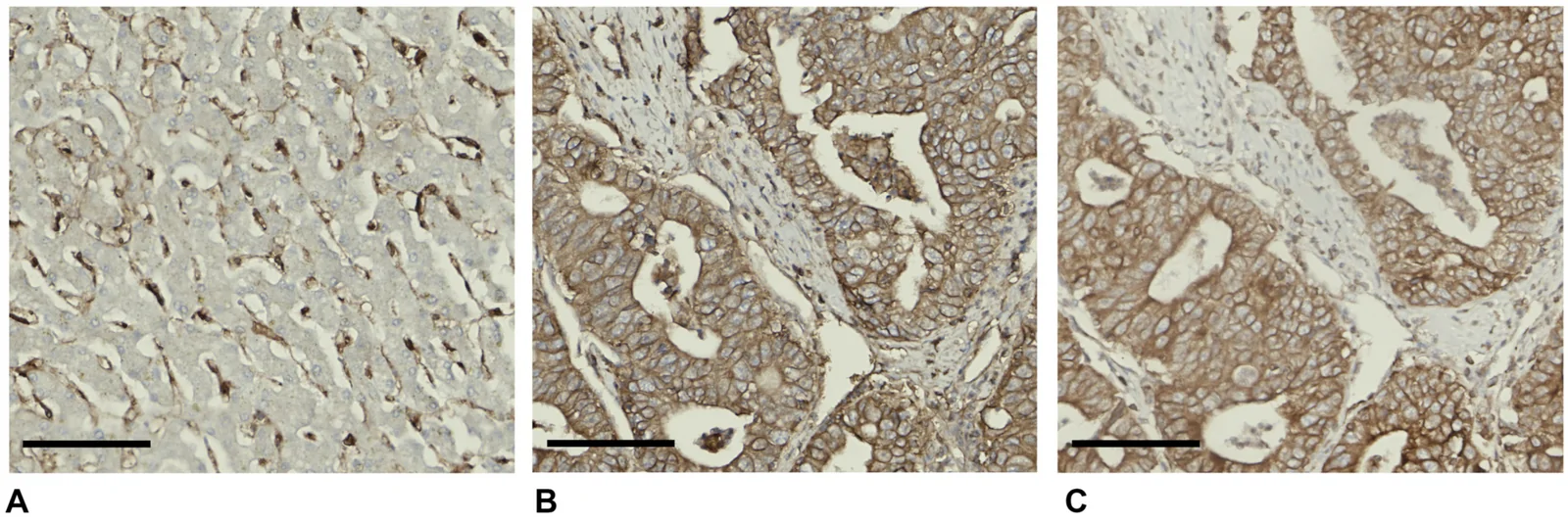
Immunohistochemical expression of HLA class I and tapasin in HCC compared with adjacent non-malignant liver tissue. (**A**) HLA class I expression in morphologically normal hepatocytes adjacent to the tumour, showing absent or minimal staining consistent with the physiological low expression of HLA class I in normal hepatic parenchyma. (**B**) HLA class I expression in HCC cells, demonstrating marked upregulation of cytoplasmic and membranous staining compared with non-tumoral tissue. (**C**) Tapasin expression in HCC tissue, showing diffuse cytoplasmic positivity in tumour cells. The upregulation of HLA class I APM components in HCC, in contrast to their absence in normal hepatocytes, supports the potential for CD8^+^ T-cell-based immunotherapeutic targeting of this tumour type. Tissue source, antibodies, staining protocol, and digitisation are as described in Figure 2. Scale bar: 100 µm.

The loss of β2-m in sHCC29 and sHCC63 was attributed to a deletion exceeding 49 kb across the β2-m gene locus, while APM component downregulation occurred at the transcriptional level. Notably, IFN-γ treatment restored the expression of some, but not all, APM components. Importantly, β2-m was also absent in the corresponding primary HCC lesions from the patients, supporting the in vivo relevance of these findings. This study is the first to report HLA-I antigen loss driven by β2-m gene deletion and APM defects in 50% (2 of 4) of the sHCC cell lines analysed. Recently, Zhang et al. [77] developed a prognostic model for HCC based on APM-related genes using Mendelian randomisation and public datasets. The model was linked to bile acid, fatty acid, and amino acid metabolism, immune infiltration, and mutations in TTN, TP53, and MUC16. Low-risk patients showed reduced immune infiltration, suggesting greater immunotherapy sensitivity. Figure 3 compares HLA class I and tapasin expression in HCC and adjacent non-malignant liver, showing minimal expression in non-tumoral parenchyma and marked upregulation in malignant cells.

### 4.5. Pancreatic Cancer

Pandha et al. [78] conducted a comprehensive investigation into the expression profiles of HLA-I, class II, and TAP in human pancreatic ductal adenocarcinoma (PDAC) tissue and 19 immortalised pancreatic cancer lines. Their analysis revealed that in tissue samples, approximately 76% of cases exhibited reduction or loss of HLA-I and TAP, with 53% showing loss or downregulation of TAP expression. Similarly, downregulation or loss of HLA-I and TAP expression was frequently observed in pancreatic cell lines. However, the study noted that reductions in class I and TAP expression were reversible when exposed to interferon-γ in vitro, suggesting a regulatory rather than structural defect in these genes. The high prevalence of HLA-I and TAP loss bears implications for immunotherapy strategies for pancreatic cancer, as such alterations could confer a selective growth advantage for malignant cells. Nonetheless, the reintroduction of expression of these molecules with cytokines, such as interferon-γ, may ultimately enable their destruction by cytotoxic T cells. Imanishi et al. [79] conducted a study on gene expression levels of APM in 13 cell lines originating from pancreatic, biliary tract, and colon cancers, utilising real-time quantitative PCR. Flow cytometric analysis revealed that pancreatic cancer cell lines, specifically MIAPaCa-2 and Panc-1, generally exhibited lower expression levels of MHC-I compared to cell lines originating from the biliary tract or colon. Their study indicates the significance of β2-m and LMP2 in mediating the expression of MHC-I across 13 GI cancer cell lines. The investigation into class I expression in 26 PDAC and 6 autologous tumour-derived cells employed a combination of immunohistochemical, biochemical, and recombinant DNA techniques [80]. The prevalence of HLA losses was found to be like that observed in other tumour types, surpassing 35%, as determined using monomorphic and locus-specific antibodies. Notably, this study unveils, for the first time, the occurrence of loss of a complete HLA haplotype in tumour tissue, proposing that this mechanism may play a role in the advancement of human cancer.

Hiraoka et al. [81] recently examined the clinicopathological significance of classical and non-classical HLA-I antigen expression in PDAC. Using IHC on 243 PDAC tissue samples, they assessed HLA-I expression and analysed its association with clinical outcomes. Notably, low HLA-I expression, observed in 33% of cases, was significantly associated with longer overall survival (OS), whereas high expression of both HLA-E and HLA-G correlated with poorer prognosis. Moreover, high HLA-I expression in PDAC cells was linked to increased expression of IFNG, which positively correlated with the expression of immune checkpoint molecules, including PD-1, PD-L1, and PD-L2. These findings suggest that elevated expression of HLA-I and non-classical HLA molecules may serve as negative prognostic markers in PDAC, potentially reflecting an IFN-γ-driven immunosuppressive microenvironment through upregulation of immune checkpoint pathways. The expression of HLA-I on PDAC cells is a potentially valuable marker for identifying patients who may benefit from immunotherapy [82]. In smaller series, reduced HLA-I expression was reported in 24% of PDAC and complete loss in 6%, without an effect on OS [83], whereas high HLA-I expression, found in 53% of cases, was associated with improved OS [82]. Interestingly, Cattaneo et al. [84] have recently evaluated the expression of B7-H3 and HLA class I and II molecules in a series of PDAC, as well as for tumour-infiltrating immune cell populations. Deficient expression of HLA-I and HLA-II was observed in 75% and 59% of samples, respectively. Loss was also locus-dependent: HLA-A was absent in 17% and heterogeneous in 58% of tumours, compared with only 4% and 55%, respectively, for HLA-B/C. Tumours retaining HLA class I expression showed significantly denser CD8^+^ and granzyme B^+^ infiltrates. However, on multivariable analysis, high membranous B7-H3 expression and low CD8^+^ T-cell density, but not HLA class I expression alone, independently predicted poor overall survival (OS; HR 2.1 for both). The key finding was the interaction between these biomarkers: defective HLA class I expression predicted shorter survival only in tumours with low B7-H3, while favourable survival was restricted to tumours combining high HLA class I with low B7-H3. Conversely, high B7-H3 expression eliminated the prognostic value of both HLA class I expression and CD8^+^ T-cell infiltration. Mechanistically, B7-H3 and HLA class I expression were positively correlated at both the mRNA and protein levels, a relationship attributed to shared transcriptional regulation by RELA. RELA showed ChIP-seq peaks at the CD276 locus, its transcript levels correlated with HLA-A, HLA-B, and HLA-C expression, and p65 silencing reduced both B7-H3 and HLA class I, but not HLA class II, expression in PDAC cell lines. These findings are consistent with NF-κB acting as a positive regulator of HLA class I, but not HLA class II, transcription. Collectively, they suggest that HLA class I expression may be a prerequisite for effective B7-H3-targeted therapy and provide a rationale for combining B7-H3 blockade with strategies that enhance HLA class I expression [84].

**Figure 4:**
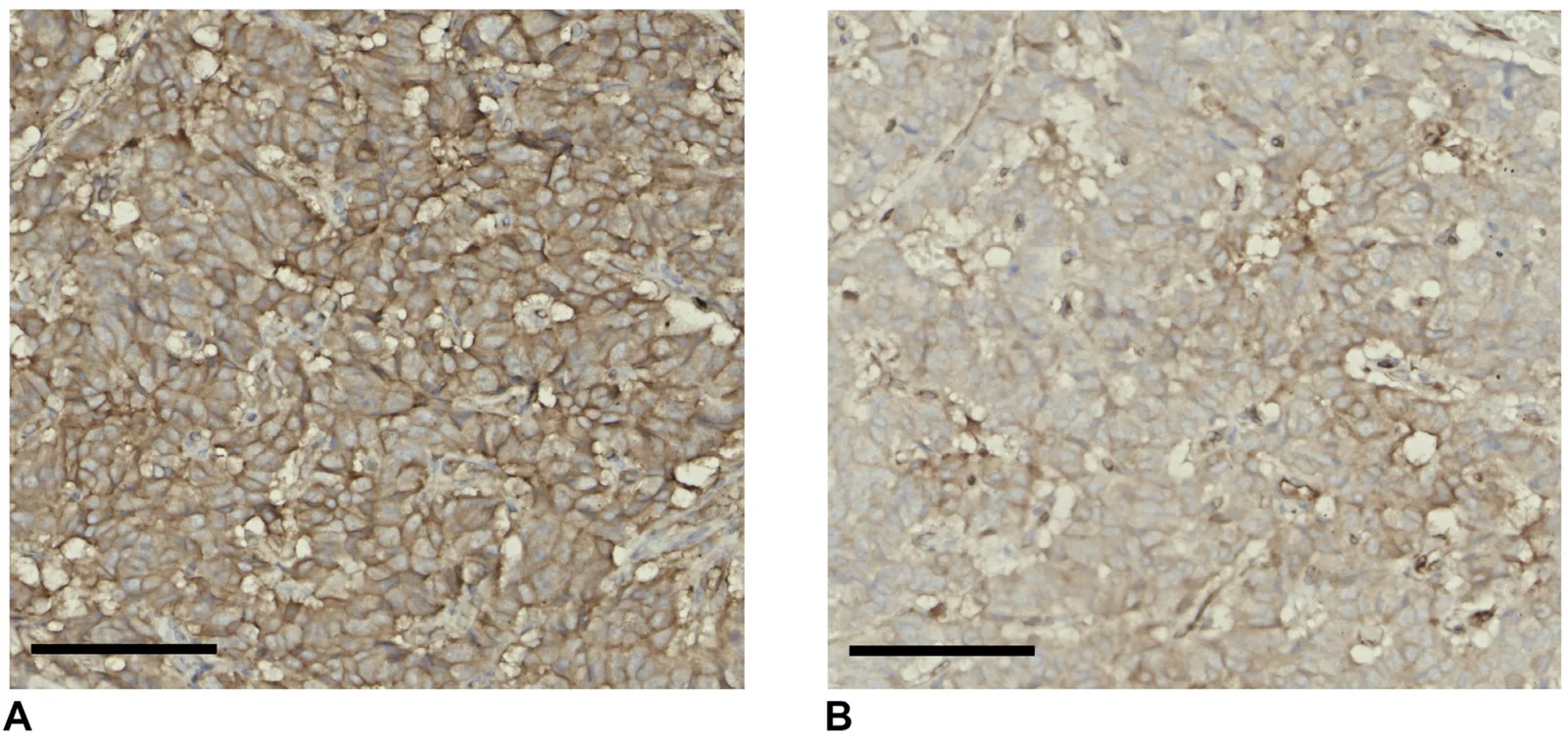
Immunohistochemical expression of HLA class I and tapasin in PDAC tissue. (**A**) HLA class I in PDAC tissue, showing heterogeneous cytoplasmic and membranous staining in malignant cells, consistent with the high frequency of HLA class I downregulation reported in this tumour type. (**B**) Tapasin expression in PDAC tissue, demonstrating variable cytoplasmic staining in tumour cells. The frequent loss or downregulation of HLA class I and tapasin in PDAC contributes to immune evasion and limits the efficacy of cytotoxic T-cell-based immunotherapies. Tissue source, antibodies, staining protocol, and digitisation are as described in Figure 2. Scale bar: 100 µm.

It has been demonstrated that radiation can amplify the immune response of cancer cells to CD8^+^ T cells by upregulating the expression of MHC-I in pancreatic cancer cells, thereby improving the efficacy of various T-cell immunotherapies [85]. Figure 4 shows heterogeneous and frequently reduced expression of both markers in malignant ductal cells, consistent with impaired antigen presentation in PDAC.

## 5. Comprehensive Bioinformatic Analysis of APM Dysregulation Across GI Cancers and Disease Progression

**Figure 5:**
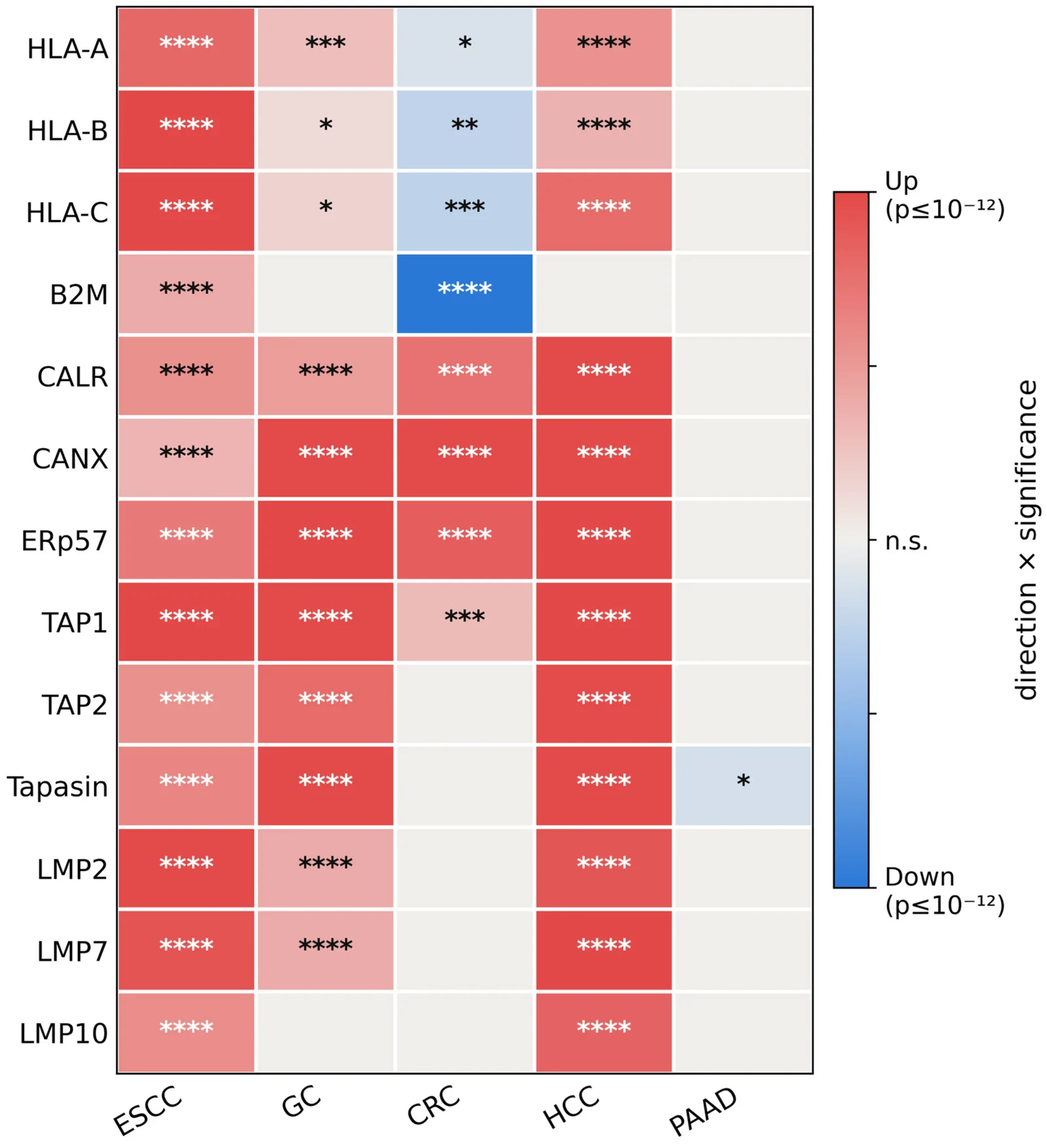
Expression of APM components in tumour versus matched normal tissue across five gastrointestinal cancers. Each cell compares tumour against normal tissue. Genes (rows): HLA-A, HLA-B, and HLA-C (HLA class I heavy chains); β2-m (β2-microglobulin); CALR (calreticulin); CANX (calnexin); ERp57 (PDIA3); TAP1 and TAP2 (transporters associated with antigen processing); Tapasin (TAPBP); immunoproteasome subunits LMP2 (PSMB9), LMP7 (PSMB8), and LMP10 (PSMB10). Cancers (columns/panels): oesophageal (ESCC), gastric (GC), colorectal (CRC), hepatocellular/liver (HCC), and pancreatic (PAAD) carcinoma. Cell colour is a continuous gradient encoding both the direction and the strength of the association: red = upregulated, blue = downregulated, and grey = not significant. Colour intensity scales with the *p*-value, using −log_10_(p) solely to map the colour axis (saturating at *p* ≤ 10^−12^). This logarithm is applied only for visualisation: the underlying expression data, the statistical tests, and all reported *p*-values are raw and untransformed, and no multiplicity/false discovery rate correction was applied. Statistical comparisons are Mann–Whitney U tests. Asterisks give the significance level of the raw *p*-value: * *p* < 0.05, ** *p* < 0.01, *** *p* < 0.001, and **** *p* < 0.0001; non-significant cells (*p* ≥ 0.05) are left grey and unmarked. The colour bar (right) runs from strong downregulation (blue, *p* ≤ 10^−12^) through non-significant (grey) to strong upregulation (red, *p* ≤ 10^−12^). The analysis was performed using the UALCAN database (https://ualcan.path.uab.edu/index.html; accessed on 16 July 2026) [86].

Figure 5 summarizes the differential expression of the major components of the APM between tumour and matched normal tissues across five gastrointestinal cancer types, providing a comprehensive overview of the direction and statistical significance of APM dysregulation in each malignancy. Because these analyses are based on bulk tissue transcriptomes, the direction of change must be interpreted in the context of the cellular composition of each tumour type and does not necessarily reflect protein expression in malignant cells, as discussed for oesophageal carcinoma in Section 4.1.

**Figure 6:**
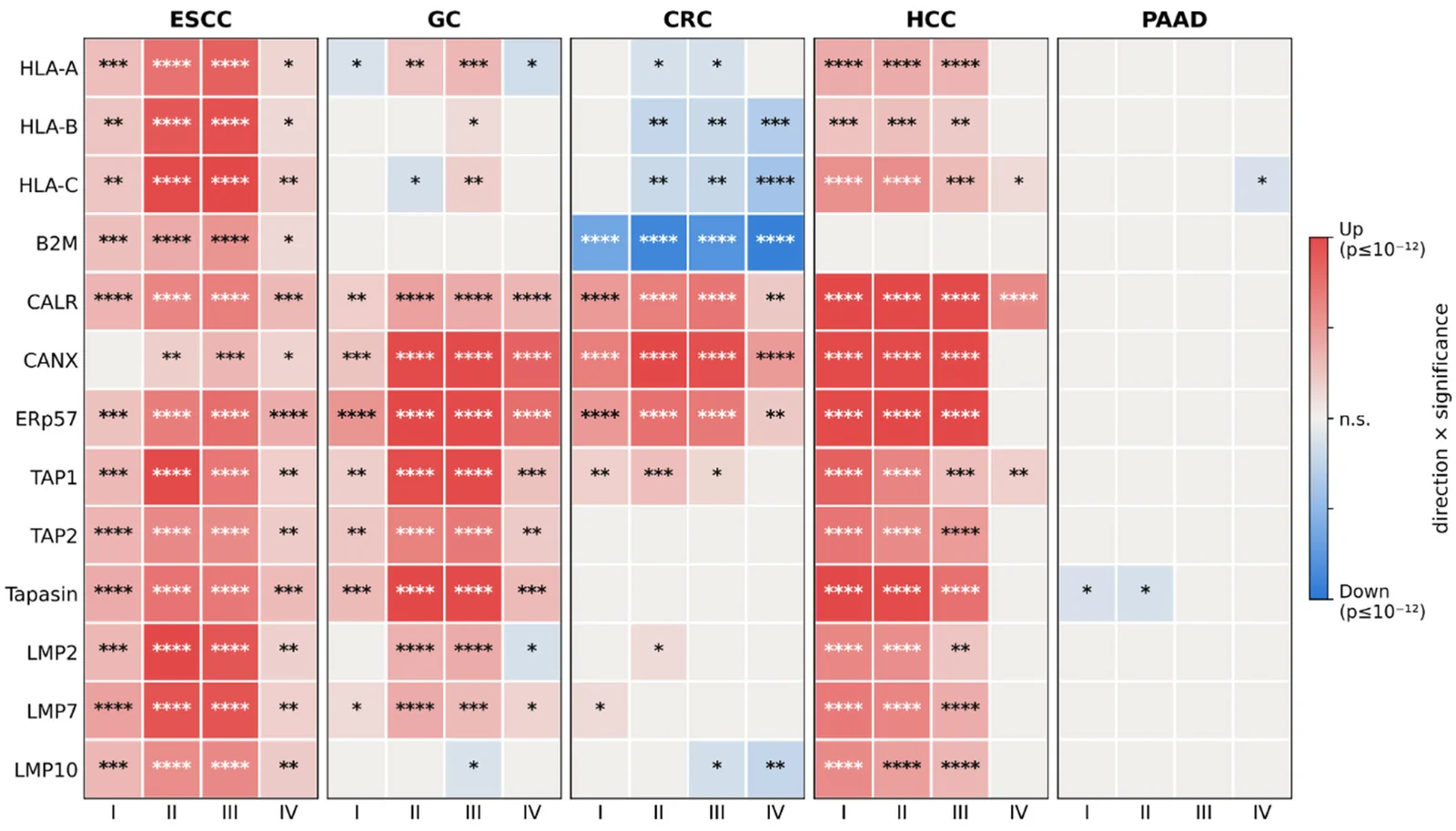
APM expression by tumour stage. Five panels (one per cancer type): within each panel the columns are AJCC tumour stages I–IV, and every cell compares that stage against normal tissue (red = upregulated vs normal, blue = downregulated vs normal). Asterisks give the significance level of the raw *p*-value: * *p* < 0.05, ** *p* < 0.01, *** *p* < 0.001, and **** *p* < 0.0001; non-significant cells (*p* ≥ 0.05) are left grey and unmarked. Genes, cancer types, statistical tests, colour scale, and colour bar are as described in Figure 5. The analysis was performed using the UALCAN database (https://ualcan.path.uab.edu/index.html; accessed on 16 July 2026) [86].

To investigate whether alterations in the APM are associated with tumour progression, we next examined the expression of individual APM components according to pathological stage. Figure 6 provides a comprehensive overview of the differential expression of APM genes across AJCC tumour stages I–IV in five GI cancer types, with each stage compared against matched normal tissue.

**Figure 7:**
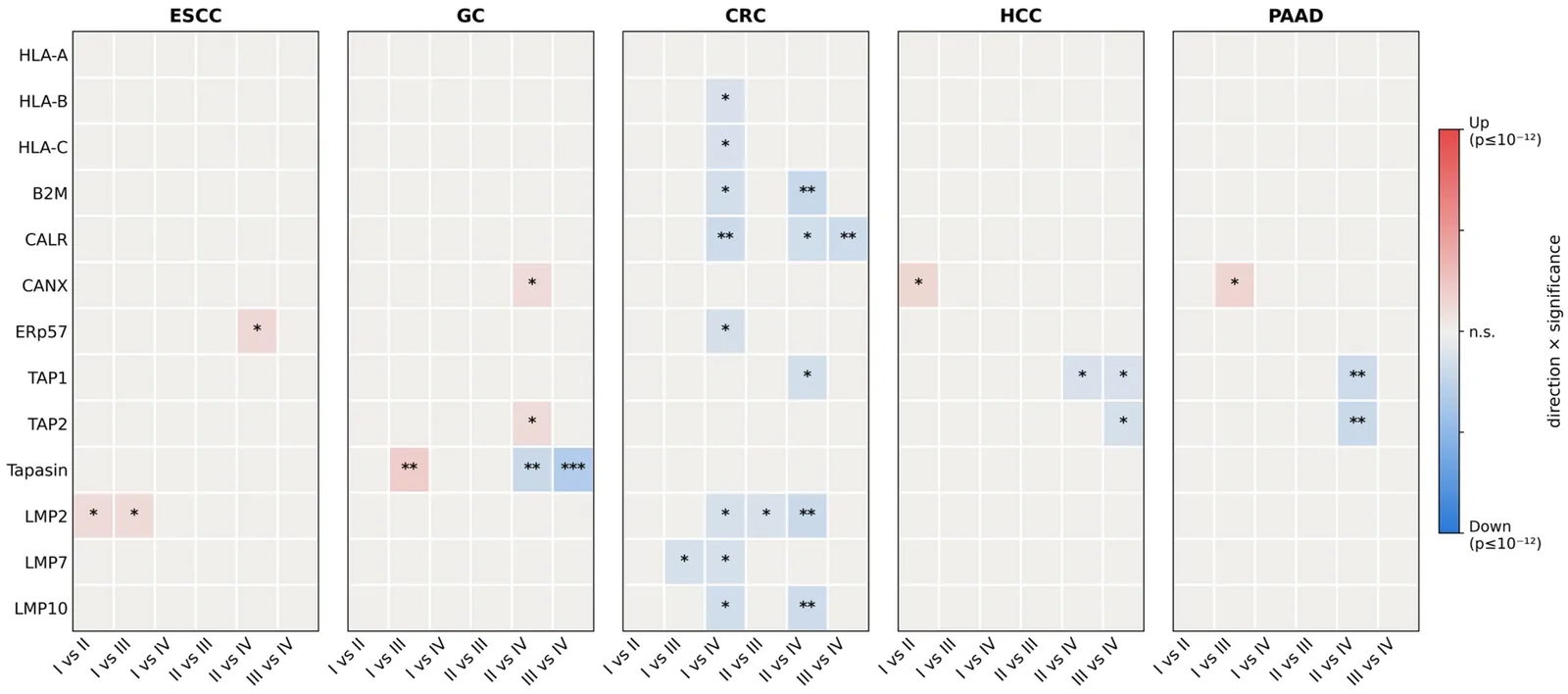
Pairwise comparisons between tumour stages (intra-stage). Columns are stage versus stage contrasts (e.g. “I vs II” = Stage I versus Stage II). Red indicates higher expression in the more advanced stage of the pair, blue indicates lower expression in the more advanced stage, and grey = not significant. Most intra-stage contrasts are non-significant, indicating that APM dysregulation is largely established at tumour onset and changes little with stage progression, the main exception being a further decline of several components in colorectal cancer. Asterisks give the significance level of the raw *p*-value: * *p* < 0.05, ** *p* < 0.01, and *** *p* < 0.001; non-significant cells (*p* ≥ 0.05) are left grey and unmarked. Genes, cancer types, statistical tests, colour scale, and colour bar are as described in Figure 5. The analysis was performed using the UALCAN database (https://ualcan.path.uab.edu/index.html; accessed on 16 July 2026) [86].

To determine whether the alterations of the APM continue to evolve during tumour progression, we next performed pairwise comparisons between consecutive and non-consecutive AJCC tumour stages within each gastrointestinal cancer type. These intra-stage contrasts, summarised in Figure 7, identify APM components that are dynamically modulated during progression rather than being established at tumour onset.

**Figure 8:**
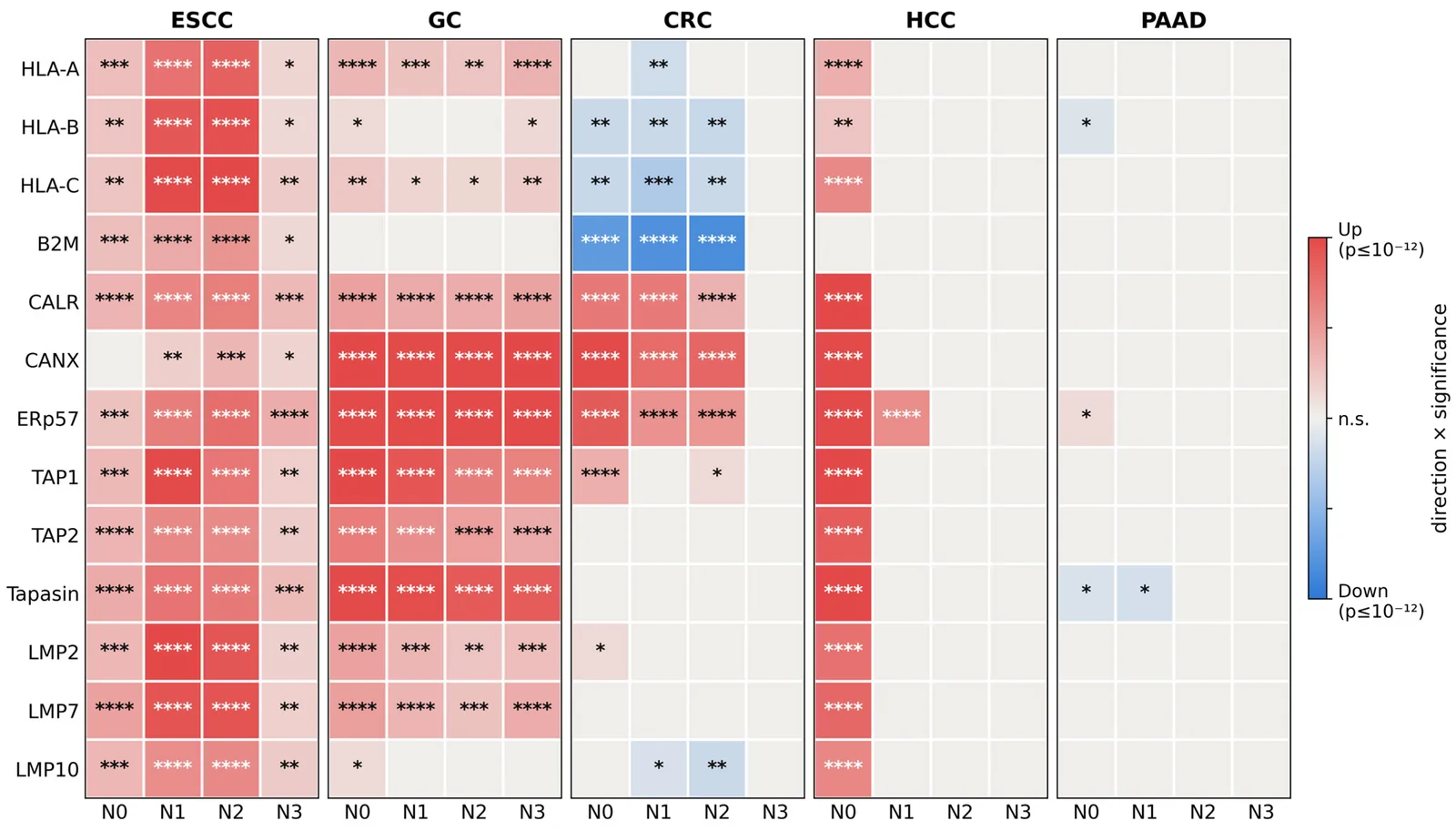
APM expression by regional lymph node status. Within each panel the columns are nodal categories N0–N3, each compared against normal tissue. Nodal data were available for the oesophageal, gastric, and colorectal datasets, while the liver and pancreatic datasets contained limited nodal information. Asterisks give the significance level of the raw *p*-value: * *p* < 0.05, ** *p* < 0.01, *** *p* < 0.001, and **** *p* < 0.0001; non-significant cells (*p* ≥ 0.05) are left grey and unmarked. Genes, cancer types, statistical tests, colour scale, and colour bar are as described in Figure 5. The analysis was performed using the UALCAN database (https://ualcan.path.uab.edu/index.html; accessed on 16 July 2026) [86].

To further explore the relationship between APM alterations and tumour aggressiveness, we investigated whether expression patterns of APM components were associated with regional lymph node involvement. Figure 8 summarizes the differential expression of APM genes across nodal categories (N0–N3) compared with matched normal tissue in gastrointestinal cancer cohorts with available lymph node information.

**Figure 9:**
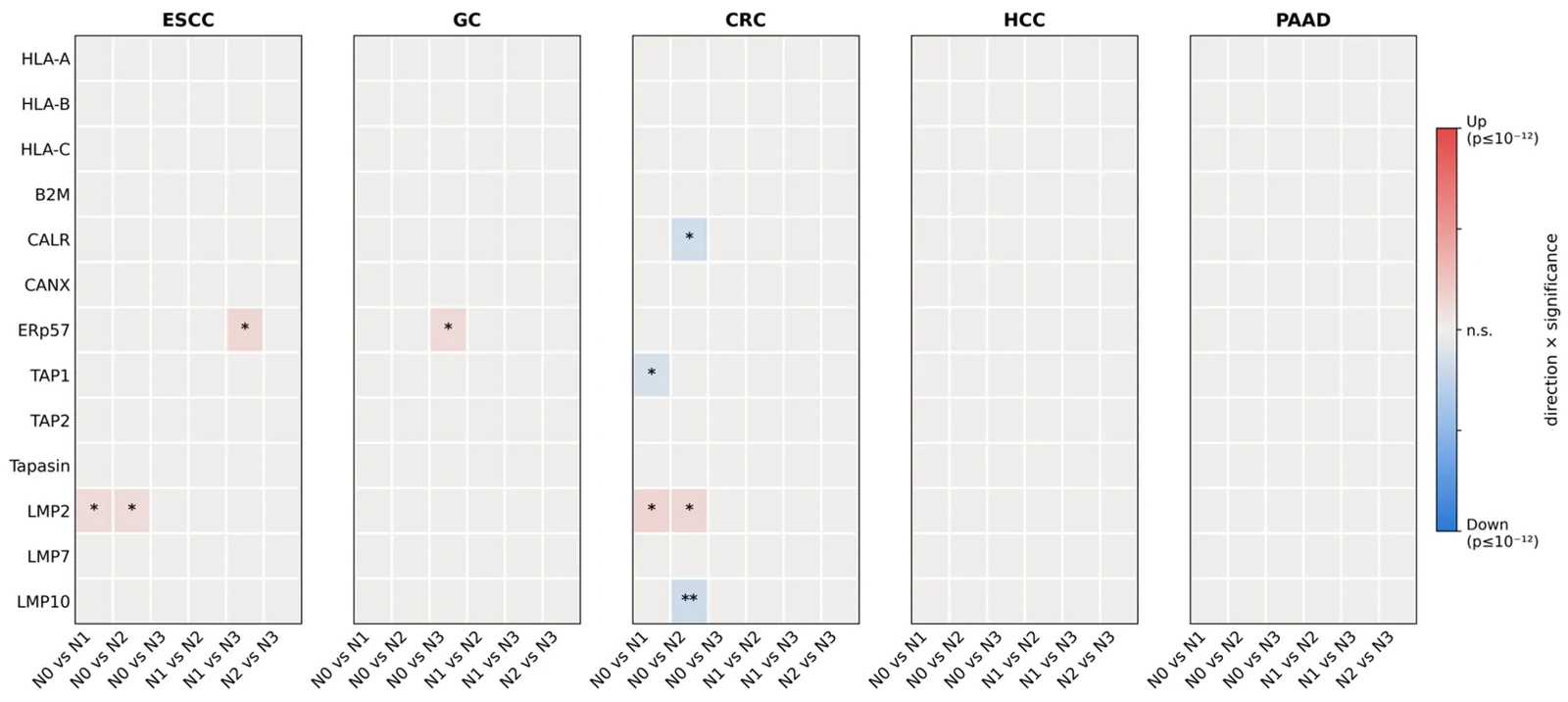
Pairwise comparisons between nodal categories (intra-nodal). Columns are node versus node contrasts (e.g. “N0 vs N1”). Red indicates higher expression in the higher nodal category of the pair, blue indicates lower expression, and grey = not significant. Asterisks give the significance level of the raw *p*-value: * *p* < 0.05, and ** *p* < 0.01; non-significant cells (*p* ≥ 0.05) are left grey and unmarked. Genes, cancer types, colour scale, statistical testing, and colour bar are as described in Figure 5. The analysis was performed using the UALCAN database (https://ualcan.path.uab.edu/index.html; accessed on 16 July 2026) [86].

To determine whether APM alterations associated with lymph node involvement represent progressive changes during metastatic dissemination or are already established in primary tumours, we performed pairwise comparisons between nodal categories. These intra-nodal contrasts are summarised in Figure 9.

## 6. MHC-I Antigen Presentation and CD8^+^ T-Cell Responses in Cancer

Defects in the APM are frequently observed across a wide range of malignancies and constitute a major mechanism of immune evasion. Tumours commonly downregulate MHC-I expression during disease progression, with an MHC-I-low phenotype reported in 40–90% of human cancers, including pancreatic cancer, non-small-cell lung cancer (NSCLC), breast cancer, prostate cancer, CRC, head and neck squamous cell carcinoma (HNSCC), HCC, and melanoma, where it is consistently associated with poor prognosis.

Loss of β2-m is a well-established mechanism of acquired resistance to immune checkpoint blockade (ICB). A longitudinal analysis of tumour biopsies from 17 patients with metastatic melanoma undergoing ICI therapies revealed point mutations, deletions, or LOH in β2-m in nearly 30% of patients with disease progression. β2-m LOH was three times more common in non-responders (29.4%) compared with responders (9.5%) [14]. Similarly, in patients with MMR-deficient colon cancer who acquired resistance to PD-1 therapy, brain metastases contained truncating β2-m mutations not present in primary tumours [14]. Importantly, disruption of IFN-γ pathway genes, including IFNGR1, IFNGR2, JAK1, and JAK2, renders cancer cells insensitive to IFN-γ, abrogating MHC-I upregulation and contributing to both primary and acquired ICI resistance across multiple tumour types [14].

Emerging evidence also highlights that IRF2, an interferon regulatory transcription factor frequently downregulated in primary cancers, acts as a transcriptional activator for immunoproteasomes, TAP, and ERAP1 [11]. When IRF2 is lost, peptide transport from the cytosol to the ER and N-terminal peptide trimming become rate-limiting steps in antigen presentation, creating an additional axis of immune escape that operates independently of classical HLA gene mutations.

Restoring MHC-I expression and APM function has emerged as a promising strategy to enhance CD8^+^ T-cell-mediated tumour immunity and improve responses to immunotherapy. In contrast to β2-m deficiency, recent studies indicate that loss of tapasin can unexpectedly promote antitumour T-cell responses and sensitise tumour cells to ICB, underscoring the complexity of antigen presentation pathways and their context-dependent roles in regulating antitumour immunity.

### 6.1. The IFN-γ–NLRC5 Axis and Transcriptional Regulation of the APM

The transcriptional regulation of HLA class I and APM genes is orchestrated by a network of master regulators, among which NLRC5 and interferon regulatory factor 1 (IRF1) play central roles. NLRC5, a non-classical MHC transactivator of the NLR family, acts as a major transcriptional coactivator for HLA class I heavy chain and several APM components, including LMP2 and LMP7 [85]. Importantly, radiation has been shown to upregulate NLRC5 in a STING- and interferon-independent manner, directly promoting MHC-I expression on cancer cells and enhancing T-cell cytotoxicity, a mechanism distinct from the classical IFN-γ pathway [85].

IFN-γ signalling drives APM upregulation primarily through JAK1/JAK2-mediated phosphorylation of STAT1, which then activates IRF1 and NLRC5 promoters. However, this pathway is frequently disrupted in cancer through loss-of-function mutations in IFNGR1, IFNGR2, JAK1, JAK2, and IRF2, as well as through epigenetic repression of their encoding genes. Loss of IFN-γ pathway genes represents a convergent mechanism of resistance to anti-CTLA-4 and anti-PD-1 therapies across multiple tumour types [87]. Conversely, when the IFN-γ pathway is intact, tumour cells respond to immune pressure by upregulating not only MHC-I and APM components but also immunosuppressive checkpoints, such as PD-L1 and PD-L2, effectively co-opting the immune response, as has been observed in PDAC [81].

This dual role of IFN-γ, promoting immune recognition while simultaneously enabling tumour adaptation, underscores the need for combination approaches that restore APM function while blocking compensatory checkpoint upregulation. The balance between these effects is highly tumour- and context-dependent, and individual APM components may respond differently to IFN-γ stimulation, necessitating multi-marker profiling to guide therapeutic decisions.

### 6.2. Beyond Genetic Defects: Post-Transcriptional, Epitranscriptomic, and Surface-Level Regulation of MHC Class I

While genomic and epigenetic mechanisms of APM disruption are well-established, a growing body of evidence highlights additional post-transcriptional, epitranscriptomic, and surface-level regulatory mechanisms that modulate MHC class I antigen presentation and contribute to immune evasion in cancer, including GI malignancies.

At the post-transcriptional level, RNA-binding proteins of the MEX3 family, particularly MEX3B, suppress HLA-A mRNA translation by binding to its 3′-untranslated region (3′-UTR), thereby reducing surface MHC-I expression independently of transcription [39]. MEX3B-mediated translational repression has been identified as a functional mechanism of resistance to ICIs in melanoma, with MEX3B expression inversely correlating with responsiveness to anti-PD-1 therapy. Similarly, microRNAs, including miR-148a-3p and miR-27a, suppress calnexin and calreticulin expression, respectively, reducing MHC-I surface assembly and exposure in CRC. At the cell surface, signal peptide peptidase-like 3 (SPPL3) alters the glycosphingolipid repertoire on the membrane of tumour cells, creating a physical phospholipid shield around peptide–MHC-I complexes that sterically inhibits TCR binding and attenuates T-cell activation despite intact surface MHC-I expression. Together, these mechanisms illustrate that even tumours maintaining apparently normal MHC-I surface levels may functionally evade T-cell recognition through post-translational interference.

At the epitranscriptomic level, N^6^-methyladenosine (m^6^A) modification, the most abundant endogenous modification of eukaryotic mRNAs, has recently emerged as an important regulator of antigen presentation. The METTL3–METTL14 methyltransferase complex deposits m^6^A on HLA-I mRNAs, promoting their translation and thus supporting surface MHC-I expression [39]. Conversely, m^6^A modification of mRNAs encoding autophagy-related genes (including ULK1, ATG5, and ATG7) recruits the reader protein YTHDF2, leading to mRNA degradation and reduced autophagy protein levels. Given that selective autophagy, mediated by NBR1, is responsible for lysosomal degradation of MHC-I trimolecular complexes in pancreatic cancer, this epitranscriptomic axis indirectly modulates MHC-I surface availability. Furthermore, in dendritic cells, m^6^A modification of lysosomal protease transcripts via YTHDF1 facilitates cross-presentation of tumour neoantigens to CD8^+^ T cells, a process relevant for the priming of antitumour T-cell responses following ICI administration. Perturbation of these epitranscriptomic circuits in cancer cells may, therefore, impair both direct antigen presentation and the cross-priming of immune responses, with implications for ICI efficacy that remain to be fully characterised in GI malignancies.

### 6.3. MHC Class I-Deficient Tumours and the NK Cell Axis: “Missing Self” Recognition and Therapeutic Exploitation

Cancers that downregulate or completely lose MHC class I expression escape CD8^+^ T-cell-mediated killing but, in so doing, become susceptible to NK cell-mediated cytotoxicity through the “missing self” mechanism [88]. NK cells are normally suppressed by inhibitory receptors recognising self-MHC-I, including KIR2D and KIR3D, CD94-NKG2A, and LILRB1. Loss of surface MHC-I relieves these inhibitory signals and, when combined with engagement of activating receptors, such as NKG2D, NKp30, NKp44, and NKp46, whose ligands are upregulated on stressed and transformed cells, triggers NK cell cytotoxicity and cytokine production [88]. This complementary immune surveillance mechanism is particularly relevant in the context of GI cancers where complete HLA-I loss drives resistance to ICI-based strategies.

However, tumours also deploy multiple strategies to evade NK cell recognition. These include (a) shedding of NKG2D ligands (soluble MICA/MICB), which competitively block NKG2D on NK cells, (b) TGF-β-mediated transcriptional suppression of NKp30 and NKG2D, and (c) metabolic exhaustion and functional anergy of intratumoral NK cells driven by chronic stimulation and immunosuppressive cytokines within the TME [88]. In GI malignancies, these NK evasion mechanisms frequently co-exist with partial or selective HLA-I loss, creating a dual immune escape phenotype that is simultaneously refractory to both CTL and NK cell surveillance.

From a therapeutic standpoint, this understanding justifies the use of NK cell-activating strategies specifically in HLA-I-deficient tumours. The anti-NKG2A monoclonal antibody monalizumab, by blocking the CD94-NKG2A inhibitory checkpoint on NK cells, restores their effector function against HLA-E-expressing MHC-I-low tumours, and has demonstrated clinical activity in combination with cetuximab in head and neck squamous cell carcinoma [39]. Additionally, bispecific antibodies targeting tumour antigens on one arm and activating NK receptors (e.g., CD16a) on the other offer a strategy to redirect NK cytotoxicity toward specific cancer cell populations regardless of HLA-I expression. In GI cancers, combinatorial strategies pairing NK cell engagers with agents that restore partial MHC-I expression, to reactivate CTL surveillance while maintaining NK activation, represent a rational and underexplored therapeutic frontier.

### 6.4. Emerging Precision Approaches: TCR-Mimetic Antibodies, PROTACs, and Integrated Biomarker Prediction

The convergence of detailed APM profiling with novel therapeutic modalities is opening new avenues for precision immuno-oncology that may prove particularly relevant for GI cancers, where heterogeneous APM defects coexist across histological subtypes. TCR-like (TCR-mimetic) antibodies, engineered to recognise specific intracellular tumour-derived peptides presented by defined HLA alleles on the tumour cell surface, overcome the dependence on T-cell priming and bypass many of the cellular immune evasion mechanisms associated with APM defects [39]. Tebentafusp, a soluble, affinity-enhanced TCR targeting the gp100 peptide–HLA-A*02:01 complex and fused to an anti-CD3 single-chain variable fragment (scFv), improved 1-year OS to 73% versus 59% in a randomised phase III trial of patients with metastatic uveal melanoma (HR 0.51, 95% CI 0.37–0.71). It was subsequently approved by the FDA in January 2022, providing clinical proof of concept for TCR-based redirection of T cells against peptide–HLA targets [39]. Analogous bispecific TCR-mimetics targeting hotspot mutations in TP53 (R175H) and RAS oncogenes, both highly prevalent in GI cancers, including colorectal, pancreatic, and gastric cancer, have demonstrated specific antitumour activity in preclinical models [39].

Proteolysis-targeting chimeras (PROTACs), heterobifunctional small molecules that recruit E3 ubiquitin ligases to degrade specific target proteins, represent another emerging strategy to modulate antigen presentation. By degrading immunosuppressive oncoproteins or proteins that facilitate MHC-I evasion, PROTACs can reshape the immunopeptidome of tumour cells, increasing the repertoire of displayed tumour antigens and potentially converting immunologically “cold” tumours to “hot” ones [39]. In GI malignancies characterised by oncogene-driven MHC-I suppression, such as KRAS-mutant pancreatic and CRCs and BRAF-mutant CRC, PROTACs targeting the driver oncogene alongside ICI may offer synergistic immunostimulatory benefit.

Finally, the integration of multiple APM-related biomarkers through machine learning approaches has demonstrated superior predictive accuracy for ICI response compared to any single parameter. A random forest model (RF16) integrating tumour mutational burden (TMB), HLA evolutionary divergence (HED), HLA-I LOH, and 13 additional genomic, demographic, and clinical features was validated in a cohort of 1479 patients across 16 cancer types and outperformed each individual biomarker [39]. HED, a measure of the physicochemical divergence between a patient’s HLA alleles, captures the breadth of the presentable peptide repertoire, is independently associated with ICI benefit across several tumour types, and interacts with TMB non-additively, since tumours with high TMB but low HED may still fail to present the resulting neoantigens. In GI malignancies, where MSI-H status (high TMB) does not uniformly predict response, incorporating HED and APM component status into predictive models represents a clinically actionable refinement toward precision immunotherapy.

## 7. Clinical Consequences and Therapeutic Strategies

### 7.1. Preclinical and Translational Evidence

Tumours with defective antigen presentation frequently show primary or acquired resistance to ICIs, and several strategies to restore or bypass APM deficiencies are under investigation [89,90]. Epigenetic therapies, such as histone deacetylase (HDAC) inhibitors or DNA methyltransferase inhibitors (DNMTi), have been shown to upregulate HLA-I and associated APM genes in cell lines and murine models. They act by increasing chromatin accessibility at APM loci and, in some cancers, by de-repressing endogenous retroviruses that trigger type I interferon production [11]. Cytokine therapies using IFN-γ or IL-12 may similarly restore antigen presentation and T-cell recruitment. These cytokine-based and epigenetic approaches, although mechanistically well established, have not yet translated into a positive randomised trial in GI cancer and should be regarded as proof-of-concept rather than as clinically validated strategies. Additionally, targeting upstream oncogenic pathways that suppress APM, such as the MAPK or Wnt/β-catenin pathways, has yielded encouraging preclinical results. Adoptive cell therapies (e.g., TCR-engineered T cells) require intact HLA presentation but may be combined with agents that restore APM function. Nearly all major classes of chemotherapeutic agents have been shown to upregulate HLA-I APM components in various cancer cell lines, both in vitro and in mouse models, thereby promoting improved recognition and elimination by antigen-specific cytotoxic CTLs. Direct clinical confirmation is, however, limited: the supporting data derive predominantly from in vitro systems and murine models, and only a minority of agents have been evaluated in paired pre- and post-treatment human biopsies [65,91,92]. Mechanistically this involves secretion of type I interferons (IFN-α/β) and activation of NF-κB signalling, which may contribute to the clinical synergy observed between chemotherapy and ICIs.

The lack of clinical documentation of radiation-induced HLA-I upregulation may in part reflect methodological limitations, as clinical studies typically compare patients who did versus did not receive radiation rather than analysing sequential biopsies from the same patient, a design that is confounded by differences in disease stage. Sequential biopsy analysis in sarcoma patients showed radiation-induced HLA class I upregulation in 60% of cases, suggesting that radiation-induced APM upregulation does occur but requires optimised dose, schedule, and timing relative to ICI administration to translate into clinical benefit [11].

An emerging strategy involves targeting aberrant trafficking of MHC class I trimolecular complexes. In PDAC, elevated autophagic flux mediated by the autophagy receptor NBR1 promotes lysosomal degradation of HLA-I complexes, and treatment with lysosome inhibitors, such as chloroquine, prevents this degradation and restores HLA-I surface expression [11]. Similarly, PCSK9 inhibition prevents cholesterol metabolism-mediated lysosomal degradation of MHC-I heavy chain, with anti-PCSK9 monoclonal antibodies (alirocumab and evolocumab, already approved for hypercholesterolaemia) showing synergy with ICIs in preclinical CRC models [11]. Both strategies are, to date, exclusively preclinical: alirocumab and evolocumab are approved for hypercholesterolaemia but have never been tested prospectively as immunosensitising agents in gastrointestinal cancer.

Beyond loss of MHC expression [5,6,7], tumours may disrupt genes involved in antigen processing and loading [93], and release immunosuppressive factors that inhibit DCs’ cross-presentation [94]. Consequently, APM dysfunction is a major determinant of poor tumour immunogenicity and limited responsiveness to immunotherapy across multiple cancer types [87].

Recently, Hu et al. [95] investigated the role of extracellular vesicles derived from activated T cells (ATEVs) in modulating APM pathways. Through characterisation of the molecular cargo and functional activity of activated T-cell extracellular vesicular DNA (AT-EVDNA), they identified a previously unrecognised mechanism that enhances tumour antigen presentation and may overcome immune evasion in immunologically “cold” cancers. Their findings reveal a transient, non-viral gene delivery strategy mediated by ATEVs that promotes APM activation and antitumour immunity while potentially limiting the risk of autoimmunity.

### 7.2. Clinical Evidence

By contrast, the evidence directly linking APM status to clinical outcomes is, at present, almost entirely observational and derives from three main sources. First, retrospective immunohistochemical series in oesophageal, gastric, colorectal, hepatocellular, and pancreatic carcinomas have consistently associated HLA class I loss with tumour grade, depth of invasion, and nodal involvement, and, in most cohorts, with shorter survival. These studies are, however, heterogeneous in their scoring criteria and sample sizes, and the direction of the association is not consistent across pancreatic and hepatocellular carcinomas. Part of this inconsistency may reflect methodological differences, but the available evidence also points to a biological explanation. In resected PDAC, defective HLA class I expression was associated with shorter survival only in tumours with low B7-H3 expression; in tumours with high B7-H3, neither HLA class I expression nor CD8^+^ T-cell density retained prognostic significance [84]. Thus, the prognostic impact of HLA class I status appears to depend on the co-expression of inhibitory checkpoint molecules. This provides a testable explanation for the variable prognostic value of HLA class I loss across cohorts and supports interpreting APM status in the context of checkpoint expression rather than as an isolated biomarker. Second, correlative analyses embedded within checkpoint inhibitor trials have shown that β2-m inactivation and HLA class I loss of heterozygosity are enriched among non-responders and in progressing lesions, supporting their role as biomarkers of acquired resistance rather than as validated treatment selection criteria. Notably, mismatch-repair-deficient tumours may retain sensitivity to checkpoint blockade despite β2-m inactivation, an effect attributed to γδ T-cell-mediated cytotoxicity [56], and pembrolizumab remains superior to chemotherapy in MSI-H metastatic colorectal cancer irrespective of APM status [53]. Third, only two agents whose mechanisms of action depend on peptide–HLA class I complexes have so far reached phase III evaluation: tebentafusp, which bypasses defective T-cell priming rather than restoring APM function, and monalizumab, which targets an inhibitory pathway involving HLA-E rather than correcting HLA class I loss. Neither has been evaluated in gastrointestinal cancer specifically in the context of APM deficiency. No prospective trial has yet used APM profiling to guide treatment allocation in gastrointestinal cancer. In our view, this represents the decisive gap between the evidence summarised above and clinical practice, and a more important unmet need than the identification of additional mechanisms of APM disruption.

## 8. Neoantigen Vaccines and the APM: Clinical Integration

The emergence of personalised neoantigen vaccines as a therapeutic modality, particularly when combined with ICIs, has created a new clinical interface between APM function and cancer immunotherapy. Deficiencies in antigen processing and presentation, including HLA-I LOH, β2-m loss, and transcriptional suppression, limit the efficacy of neoantigen vaccines by reducing the presentation of vaccine-encoded epitopes on tumour cell surfaces. In PDAC, personalised RNA neoantigen vaccines targeting up to 20 patient-specific neoantigens have shown promise, with de novo neoantigen-specific T-cell responses associated with delayed recurrence in 8 of 16 vaccinated patients [14]. These advances underscore the critical importance of accounting for APM defects in vaccine design: neoantigens predicted to bind exclusively to HLA alleles lost through LOH should be excluded from vaccination strategies, as they cannot be presented by tumour cells and may drive selective pressure toward clones lacking those alleles [14]. Vaccine design, therefore, requires integrated genomic, transcriptomic, and immunopeptidomic profiling, in order to identify HLA-I/II LOH and to prioritise clonal neoantigens restricted to retained alleles. End-to-end proteogenomic pipelines, such as NeoDisc, implement these criteria and are expected to accelerate the clinical implementation of personalised cancer vaccines [14]. This matters particularly in GI malignancies, where several patterns of APM dysfunction coexist.

## 9. Conclusions

Disruption of the HLA-I APM is a key mechanism of immune evasion and a significant obstacle to effective immunotherapy in GI cancers.

The central message of this review is that, in GI cancer, the APM should be considered a therapeutic variable rather than merely a descriptive feature. HLA class I loss is not a uniform phenomenon: it affects approximately 30–76% of tumours, depending on tumour site and molecular subtype, it arises through mechanistically distinct pathways, and, importantly, only some of these alterations are potentially reversible. We, therefore, propose that patients should be stratified not simply according to the presence or absence of HLA class I staining, but according to the mechanism underlying its loss. Reversible defects, including epigenetic and transcriptional silencing, post-transcriptional repression, and aberrant trafficking, and irreversible alterations, such as β2-m mutation, HLA class I loss of heterozygosity, and disruption of IFN-γ signalling, call for distinct therapeutic strategies: restoration of antigen presentation in the former and circumvention of defective antigen presentation in the latter.

We do not consider interferons and HDAC inhibitors to represent the most promising therapeutic frontier. Their ability to increase HLA class I expression has been recognised for decades, the effect may be transient and limited by toxicity, and IFN-γ simultaneously induces PD-L1, PD-L2, and the non-classical HLA molecules HLA-E and HLA-G, potentially increasing antigen presentation and immunoregulatory signalling in parallel. These agents may, therefore, be most useful as conditioning strategies in combination with immune checkpoint blockade rather than as standalone approaches. Four therapeutic directions appear particularly promising. First, mechanism-informed neoantigen vaccination: HLA class I loss of heterozygosity should be assessed before epitope selection, because epitopes restricted by lost HLA alleles are unlikely to be presented and may impose selective pressure favouring immune escape. End-to-end proteogenomic pipelines make such an approach increasingly feasible. Second, redirection of cytotoxicity independently of endogenous T-cell priming through soluble TCR-based bispecifics, including ImmTACs, and TCR-mimetic antibodies directed against shared driver-derived epitopes, such as KRAS G12D and TP53 R175H. Third, therapeutic exploitation of innate immune recognition in HLA class I-deficient tumours, including approaches targeting the missing-self response through NK cell engagers, CD16a engagement, NKG2A/HLA-E axis modulation, and adoptive NK cell therapies. The rationale for these strategies is particularly relevant where defective antigen presentation limits conventional T-cell-mediated recognition, although their clinical efficacy remains to be established in this setting. Fourth, restoration of cell-surface peptide–HLA complexes by targeting post-translational mechanisms of degradation, including NBR1-dependent autophagic degradation. In PDAC, this pathway has been shown to reduce surface MHC-I expression, while inhibition of autophagy can restore MHC-I surface levels and antigen presentation in preclinical models.

Realising these opportunities requires a diagnostic capability that is not yet established in routine pathology: a validated, quantitative, cell-resolved assessment of APM status that integrates multiplex immunofluorescence with allele-specific HLA typing and, where available, immunopeptidomics. As illustrated by our analysis of oesophageal carcinoma, bulk transcriptomic surrogates may diverge from protein-level findings and, therefore, cannot substitute for cell-resolved assessment of APM expression. We, therefore, recommend that APM profiling be incorporated prospectively as a stratification factor in the next generation of immunotherapy trials in gastrointestinal cancer. Until such studies are undertaken, the mechanistic understanding of APM disruption will continue to expand without necessarily translating into changes in patient selection or treatment.

## Data Availability

No new data were created.

## Abbreviations

The following abbreviations are used in this manuscript:

AJCC	American Joint Committee on Cancer
APM	Antigen Processing and Presentation Machinery
AT-EVDNA/ATEV	activated T-cell extracellular vesicular DNA/vesicle
β2-m	beta-2-microglobulin
B7-H3	B7 homolog 3 (CD276)
BRAF	B-Raf proto-oncogene
CALR	calreticulin
CANX	calnexin
CD8+	cluster of differentiation 8-positive (T cell)
CD94/NKG2A	natural killer cell inhibitory receptor complex
CI	confidence interval
CIITA	class II major histocompatibility complex transactivator
CIR	cancer immune responsiveness
cMS	coding microsatellites
CpG	cytosine–phosphate–guanine dinucleotide
CRC	colorectal cancer
CTL	cytotoxic T lymphocyte
CTLA-4	cytotoxic T-lymphocyte-associated protein 4
DC	dendritic cell
dMMR/MMR-d	deficient mismatch repair
DNMTi	DNA methyltransferase inhibitor
ER	endoplasmic reticulum
ERAP1/ERAP2	endoplasmic reticulum aminopeptidase 1/2
ERp57 (PDIA3)	endoplasmic reticulum protein 57/protein disulfide isomerase family A member 3
ESCC	oesophageal squamous cell carcinoma
EZH2	enhancer of zeste homolog 2
FDA	United States Food and Drug Administration
FSP	frameshift peptide
GC	gastric cancer
GI	gastrointestinal
GLOBOCAN	Global Cancer Observatory database
HCC	hepatocellular carcinoma
HDAC	histone deacetylase
HED HLA	evolutionary divergence
HLA	human leukocyte antigen
HLA-I/HLA-II	human leukocyte antigen class I/class II
HLA-A, -B, -C	classical HLA class I molecules
HLA-E, -F, -G	non-classical HLA class I molecules
HLA-DR	HLA class II DR isotype
HLA-HC/HLA-I HC	HLA class I heavy chain
HNSCC	head and neck squamous cell carcinoma
HPV16	human papillomavirus type 16
HR	hazard ratio
ICB	immune checkpoint blockade
ICI	immune checkpoint inhibitor
IFN-α/β	type I interferons
IFN-γ (IFNG)	interferon gamma
IFNGR1/IFNGR2	interferon gamma receptor 1/2
IHC	immunohistochemistry
IL-12	interleukin 12
ImmTAC	immune-mobilizing monoclonal T-cell receptor against cancer
IRF1/IRF2	interferon regulatory factor 1/2
JAK1/JAK2	Janus kinase 1/2
KIR2D/KIR3D	killer-cell immunoglobulin-like receptors (two/three domains)
KRAS	Kirsten rat sarcoma viral oncogene homolog
LILRB1	leukocyte immunoglobulin-like receptor B1
LMP2/LMP7/LMP10	immunoproteasome subunits (PSMB9/PSMB8/PSMB10)
LOH	loss of heterozygosity
m^6^A	N^6^-methyladenosine
MAPK	mitogen-activated protein kinase
MEX3B	mex-3 RNA-binding family member B
MHC	major histocompatibility complex
MHC-I	major histocompatibility complex class I
MHC-I-HC	MHC class I heavy chain
MICA/MICB	MHC class I chain-related protein A/B
MMR	mismatch repair
MP-FL	multiplex fluorescence
mRNA	messenger RNA
MSI-H	microsatellite instability-high
MSS	microsatellite stable
NBR1	neighbour of BRCA1 gene 1 (autophagy receptor)
NF-κB	nuclear factor kappa B
NK	natural killer (cell)
NKG2D	natural killer group 2 member D
NKp30/NKp44/NKp46	natural cytotoxicity-triggering receptors
NLRC5	NLR family CARD domain-containing protein 5
NSCLC	non-small-cell lung cancer
OS	overall survival
PAAD	pancreatic adenocarcinoma (TCGA project code)
PCSK9	proprotein convertase subtilisin/kexin type 9
PDAC	pancreatic ductal adenocarcinoma
PD-1	programmed cell death protein 1
PD-L1/PD-L2	programmed death ligand 1/2
PLC	peptide-loading complex
PRC2	polycomb repressive complex 2
PROTAC	proteolysis-targeting chimera
PSMB8/PSMB9/PSMB10	proteasome 20S subunit beta 8/9/10
RF16	random forest model integrating 16 features
RNA-seq	RNA sequencing
scFv	single-chain variable fragment
sHCC	sarcomatoid hepatocellular carcinoma
sHLA	soluble HLA
SITC	Society for Immunotherapy of Cancer
SPPL3	signal peptide peptidase-like 3
STAT1	signal transducer and activator of transcription 1
STING	stimulator of interferon genes
STORMI	Standards for Reporting of Multiplex Immunohistochemistry/Immunofluorescence
TAP1/TAP2	transporters associated with antigen processing 1/2
TAPBP	tapasin (TAP-binding protein)
TCR	T-cell receptor
TGF-β	transforming growth factor beta
TIL	tumour-infiltrating lymphocyte
TMB	tumour mutational burden
TME	tumour microenvironment
UTR	untranslated region
Wnt/β-catenin	Wnt/β-catenin signalling pathway
YTHDF1/YTHDF2	YTH N6-methyladenosine RNA-binding proteins 1/2

## References

[1] Hiam-Galvez K.J., Allen B.M., Spitzer M.H. (2021). Systemic immunity in cancer. Nat. Rev. Cancer.

[2] Gonzalez H., Hagerling C., Werb Z. (2018). Roles of the immune system in cancer: From tumor initiation to metastatic progression. Genes Dev..

[3] Garrido F., Aptsiauri N. (2019). Cancer immune escape: MHC expression in primary tumours versus metastases. Immunology.

[4] Danelli L. (2024). Overcoming immunoevasion in MHC class I-deficient cancers. Nat. Cancer.

[5] Cornel A.M., Mimpen I.L., Nierkens S. (2020). MHC Class I Downregulation in Cancer: Underlying Mechanisms and Potential Targets for Cancer Immunotherapy. Cancers.

[6] Wang J., Lu Q., Chen X., Aifantis I. (2024). Targeting MHC-I inhibitory pathways for cancer immunotherapy. Trends Immunol..

[7] Hicklin D.J., Marincola F.M., Ferrone S. (1999). HLA class I antigen downregulation in human cancers: T-cell immunotherapy revives an old story. Mol. Med. Today.

[8] Maggs L., Sadagopan A., Moghaddam A.S., Ferrone S. (2021). HLA class I antigen processing machinery defects in antitumor immunity and immunotherapy. Trends Cancer.

[9] Seliger B., Kloor M., Ferrone S. (2017). HLA class II antigen-processing pathway in tumors: Molecular defects and clinical relevance. OncoImmunology.

[10] Cai L., Michelakos T., Yamada T., Fan S., Wang X., Schwab J.H., Ferrone C.R., Ferrone S. (2018). Defective HLA class I antigen processing machinery in cancer. Cancer Immunol. Immunother..

[11] Sadagopan A., Michelakos T., Boyiadzis G., Ferrone C., Ferrone S. (2022). Human Leukocyte Antigen Class I Antigen-Processing Machinery Upregulation by Anticancer Therapies in the Era of Checkpoint Inhibitors. JAMA Oncol..

[12] Garrido F. (2019). MHC/HLA Class I Loss in Cancer Cells. MHC Class-I Loss and Cancer Immune Escape.

[13] Hazini A., Fisher K., Seymour L. (2021). Deregulation of HLA-I in cancer and its central importance for immunotherapy. J. Immunother. Cancer.

[14] Huber F., Bassani-Sternberg M. (2025). Defects in antigen processing and presentation: Mechanisms, immune evasion and implications for cancer vaccine development. Nat. Rev. Immunol..

[15] Lawand M., Al Rayess S., Jaber R., van Endert P. (2026). Messing with Signal 1: How Perturbed MHC Class I Antigen Presentation Contributes to Cancer. Cells.

[16] Blees A., Januliene D., Hofmann T., Koller N., Schmidt C., Trowitzsch S., Moeller A., Tampé R. (2017). Structure of the human MHC-I peptide-loading complex. Nature.

[17] Vassilakos A., Cohen-Doyle M.F., Peterson P.A., Jackson M.R., Williams D.B. (1996). The molecular chaperone calnexin facilitates folding and assembly of class I histocompatibility molecules. EMBO J..

[18] Purcell A.W., Gorman J.J., Garcia-Peydró M., Paradela A., Burrows S.R., Talbo G.H., Laham N., Peh C.A., Reynolds E.C., López de Castro J.A. (2001). Quantitative and qualitative influences of tapasin on the class I peptide repertoire. J. Immunol..

[19] Kanaseki T., Lind K.C., Escobar H., Nagarajan N., Reyes-Vargas E., Rudd B., Rockwood A.L., Van Kaer L., Sato N., Delgado J.C. (2013). ERAAP and Tapasin Independently Edit the Amino and Carboxyl Termini of MHC Class I Peptides. J. Immunol..

[20] Fucikova J., Spisek R., Kroemer G., Galluzzi L. (2020). Calreticulin and cancer. Cell Res..

[21] Parra E.R. (2021). Methods to Determine and Analyze the Cellular Spatial Distribution Extracted from Multiplex Immunofluorescence Data to Understand the Tumor Microenvironment. Front. Mol. Biosci..

[22] Sater S., Bifulco C.B., Rodriguez-Canales J., Yeong J., Akturk G., Angelo M., Ballesteros-Merino C., Bankhead P., Basu S., Blando J.M. (2025). Society for Immunotherapy of Cancer: Standards for Reporting of Multiplex Immunohistochemistry/Immunofluorescence Assays (STORMI). J. Immunother. Cancer.

[23] Parra E.R., Jiang M., Solis L., Mino B., Laberiano C., Hernandez S., Gite S., Verma A., Tetzlaff M., Haymaker C. (2020). Procedural Requirements and Recommendations for Multiplex Immunofluorescence Tyramide Signal Amplification Assays to Support Translational Oncology Studies. Cancers.

[24] Barua S., Solis L., Parra E.R., Uraoka N., Jiang M., Wang H., Rodriguez-Canales J., Wistuba I., Maitra A., Sen S. (2018). A Functional Spatial Analysis Platform for Discovery of Immunological Interactions Predictive of Low-Grade to High-Grade Transition of Pancreatic Intraductal Papillary Mucinous Neoplasms. Cancer Inform..

[25] Yu C.C., Wortman J.C., He T.-F., Solomon S., Zhang R.Z., Rosario A., Wang R., Tu T.Y., Schmolze D., Yuan Y. (2021). Physics approaches to the spatial distribution of immune cells in tumors. Rep. Prog. Phys..

[26] Grizzi F., Chiriva-Internati M., Bresalier R.S., Taverna G., Pasqualini F., Hegazi M.A.A.A. (2024). Investigating the Spatial Distribution of Proliferating Cells in Primary Ovarian Cancers. Discov. Med..

[27] Anichini A., Mortarini R., Nonaka D., Molla A., Vegetti C., Montaldi E., Wang X., Ferrone S. (2006). Association of antigen-processing machinery and HLA antigen phenotype of melanoma cells with survival in American Joint Committee on Cancer stage III and IV melanoma patients. Cancer Res..

[28] MacDonald W.J., Purcell C., Pinho-Schwermann M., Stubbs N.M., Srinivasan P.R., El-Deiry W.S. (2025). Heterogeneity in Cancer. Cancers.

[29] Seliger B., Atkins D., Bock M., Ritz U., Ferrone S., Huber C., Störkel S. (2003). Characterization of human lymphocyte antigen class I antigen-processing machinery defects in renal cell carcinoma lesions with special emphasis on transporter-associated with antigen-processing down-regulation. Clin. Cancer Res..

[30] Sung H., Ferlay J., Siegel R.L., Laversanne M., Soerjomataram I., Jemal A., Bray F. (2021). Global Cancer Statistics 2020: GLOBOCAN Estimates of Incidence and Mortality Worldwide for 36 Cancers in 185 Countries. CA Cancer J. Clin..

[31] Sheyhidin I., Hasim A., Zheng F., Ma H. (2015). Epigenetic Changes within the Promoter Regions of Antigen Processing Machinery Family Genes in Kazakh Primary Esophageal Squamous Cell Carcinoma. Asian Pac. J. Cancer Prev..

[32] Jensen P.E. (2007). Recent advances in antigen processing and presentation. Nat. Immunol..

[33] Tanaka K., Tsuchikawa T., Miyamoto M., Maki T., Ichinokawa M., Kubota K.C., Shichinohe T., Hirano S., Ferrone S., Dosaka-Akita H. (2012). Down-regulation of Human Leukocyte Antigen class I heavy chain in tumors is associated with a poor prognosis in advanced esophageal cancer patients. Int. J. Oncol..

[34] Liu Q., Hao C., Su P., Shi J. (2009). Down-regulation of HLA class I antigen-processing machinery components in esophageal squamous cell carcinomas: Association with disease progression. Scand. J. Gastroenterol..

[35] Ayshamgul H., Ma H., Ilyar S., Zhang L.W., Abulizi A. (2011). Association of defective HLA-I expression with antigen processing machinery and their association with clinicopathological characteristics in Kazak patients with esophageal cancer. Chin. Med. J..

[36] Zhang X., Lin A., Zhang J.-G., Bao W.-G., Xu D.-P., Ruan Y.-Y., Yan W.-H. (2012). Alteration of HLA-F and HLA I antigen expression in the tumor is associated with survival in patients with esophageal squamous cell carcinoma. Int. J. Cancer.

[37] Mizukami Y., Kono K., Maruyama T., Watanabe M., Kawaguchi Y., Kamimura K., Fujii H. (2008). Downregulation of HLA Class I molecules in the tumour is associated with a poor prognosis in patients with oesophageal squamous cell carcinoma. Br. J. Cancer.

[38] Shao S., Pan C., Ni Q., Huang J. (2025). Radiation regulates the expression of HLA-I in esophageal squamous cell carcinoma through interferon-gamma. Sci. Rep..

[39] Yang K., Halima A., Chan T.A. (2023). Antigen presentation in cancer—Mechanisms and clinical implications for immunotherapy. Nat. Rev. Clin. Oncol..

[40] Joshi S.S., Badgwell B.D. (2021). Current treatment and recent progress in gastric cancer. CA Cancer J. Clin..

[41] Mimura K., Shiraishi K., Mueller A., Izawa S., Kua L.-F., So J., Yong W.-P., Fujii H., Seliger B., Kiessling R. (2013). The MAPK Pathway Is a Predominant Regulator of HLA-A Expression in Esophageal and Gastric Cancer. J. Immunol..

[42] Hirata T., Yamamoto H., Taniguchi H., Horiuchi S., Oki M., Adachi Y., Imai K., Shinomura Y. (2007). Characterization of the immune escape phenotype of human gastric cancers with and without high-frequency microsatellite instability. J. Pathol..

[43] López-Nevot M.A., Esteban F., Ferrón A., Gutiérrez J., Oliva M.R., Romero C., Huelin C., Ruiz-Cabello F., Garrido F. (1989). HLA class I gene expression on human primary tumours and autologous metastases: Demonstration of selective losses of HLA antigens on colorectal, gastric and laryngeal carcinomas. Br. J. Cancer.

[44] Wang X.C., Zhang J.Q., Shen Y.Q., Miao F.Q., Xie W. (2006). Loss of heterozygosity at 6p21.3 underlying HLA class I downregulation in gastric cancer. J. Exp. Clin. Cancer Res..

[45] Satoh A., Toyota M., Ikeda H., Morimoto Y., Akino K., Mita H., Suzuki H., Sasaki Y., Kanaseki T., Takamura Y. (2004). Epigenetic inactivation of class II transactivator (CIITA) is associated with the absence of interferon-γ-induced HLA-DR expression in colorectal and gastric cancer cells. Oncogene.

[46] Yoshii M., Tanaka H., Ohira M., Muguruma K., Sakurai K., Kubo N., Yashiro M., Sawada T., Hirakawa K. (2013). Association of MHC class I expression and lymph node metastasis of gastric carcinoma. Hepatogastroenterology.

[47] Cabrera C.M., Jiménez P., Cabrera T., Esparza C., Ruiz-Cabello F., Garrido F. (2003). Total loss of MHC class I in colorectal tumors can be explained by two molecular pathways: β2-microglobulin inactivation in MSI-positive tumors and LMP7/TAP2 downregulation in MSI-negative tumors. Tissue Antigens.

[48] Atkins D., Breuckmann A., Schmahl G.E., Binner P., Ferrone S., Krummenauer F., Störkel S., Seliger B. (2003). MHC class I antigen processing pathway defects, ras mutations and disease stage in colorectal carcinoma. Int. J. Cancer.

[49] Kloor M., Becker C., Benner A., Woerner S.M., Gebert J., Ferrone S., von Knebel Doeberitz M. (2005). Immunoselective Pressure and Human Leukocyte Antigen Class I Antigen Machinery Defects in Microsatellite Unstable Colorectal Cancers. Cancer Res..

[50] Dierssen J.W.F., de Miranda N.F.C.C., Ferrone S., van Puijenbroek M., Cornelisse C.J., Fleuren G.J., van Wezel T., Morreau H. (2007). HNPCC versus sporadic microsatellite-unstable colon cancers follow different routes toward loss of HLA class I expression. BMC Cancer.

[51] Bolzacchini E., Libera L., Church S.E., Sahnane N., Bombelli R., Digiacomo N., Giordano M., Petracco G., Sessa F., Capella C. (2022). Tumor Antigenicity and a Pre-Existing Adaptive Immune Response in Advanced BRAF Mutant Colorectal Cancers. Cancers.

[52] Kloor M., von Knebel Doeberitz M. (2016). The Immune Biology of Microsatellite-Unstable Cancer. Trends Cancer.

[53] André T., Shiu K.-K., Kim T.W., Jensen B.V., Jensen L.H., Punt C.J.A., Smith D., Garcia-Carbonero R., Benavides M., Gibbs P. (2020). Pembrolizumab in Microsatellite-Instability–High Advanced Colorectal Cancer. N. Engl. J. Med..

[54] Grasso C.S., Giannakis M., Wells D.K., Hamada T., Mu X.J., Quist M., Nowak J.A., Nishihara R., Qian Z.R., Inamura K. (2018). Genetic Mechanisms of Immune Evasion in Colorectal Cancer. Cancer Discov..

[55] Kobayashi Y., Niida A., Nagayama S., Saeki K., Haeno H., Takahashi K.K., Hayashi S., Ozato Y., Saito H., Hasegawa T. (2023). Subclonal accumulation of immune escape mechanisms in microsatellite instability-high colorectal cancers. Br. J. Cancer.

[56] de Vries N.L., van de Haar J., Veninga V., Chalabi M., Ijsselsteijn M.E., van der Ploeg M., van den Bulk J., Ruano D., van den Berg J.G., Haanen J.B. (2023). γδ T cells are effectors of immunotherapy in cancers with HLA class I defects. Nature.

[57] Watson N.F.S., Ramage J.M., Madjd Z., Spendlove I., Ellis I.O., Scholefield J.H., Durrant L.G. (2005). Immunosurveillance is active in colorectal cancer as downregulation but not complete loss of MHC class I expression correlates with a poor prognosis. Int. J. Cancer.

[58] Speetjens F.M., de Bruin E.C., Morreau H., Zeestraten E.C.M., Putter H., van Krieken J.H., van Buren M.M., van Velzen M., Dekker-Ensink N.G., van de Velde C.J.H. (2007). Clinical impact of HLA class I expression in rectal cancer. Cancer Immunol. Immunother..

[59] Madjd Z., Spendlove I., Pinder S.E., Ellis I.O., Durrant L.G. (2005). Total loss of MHC class I is an independent indicator of good prognosis in breast cancer. Int. J. Cancer.

[60] Mehta A.M., Jordanova E.S., Kenter G.G., Ferrone S., Fleuren G.-J. (2007). Association of antigen processing machinery and HLA class I defects with clinicopathological outcome in cervical carcinoma. Cancer Immunol. Immunother..

[61] Djajadiningrat R.S., Horenblas S., Heideman D.A.M., Sanders J., de Jong J., Jordanova E.S. (2015). Classic and Nonclassic HLA Class I Expression in Penile Cancer and Relation to HPV Status and Clinical Outcome. J. Urol..

[62] Michelakos T., Kontos F., Kurokawa T., Cai L., Sadagopan A., Krijgsman D., Weichert W., Durrant L.G., Kuppen P.J.K., Ferrone C.R. (2022). Differential role of HLA-A and HLA-B, C expression levels as prognostic markers in colon and rectal cancer. J. Immunother. Cancer.

[63] Sers C., Kuner R., Falk C.S., Lund P., Sueltmann H., Braun M., Buness A., Ruschhaupt M., Conrad J., Mang-Fatehi S. (2009). Down-regulation of HLA Class I and NKG2D ligands through a concerted action of MAPK and DNA methyltransferases in colorectal cancer cells. Int. J. Cancer.

[64] Ozcan M., Janikovits J., von Knebel Doeberitz M., Kloor M. (2018). Complex pattern of immune evasion in MSI colorectal cancer. OncoImmunology.

[65] Wan S., Pestka S., Jubin R.G., Lyu Y.L., Tsai Y.-C., Liu L.F. (2012). Chemotherapeutics and Radiation Stimulate MHC Class I Expression through Elevated Interferon-beta Signaling in Breast Cancer Cells. PLoS ONE.

[66] Ranoa D.R.E., Parekh A.D., Pitroda S.P., Huang X., Darga T., Wong A.C., Huang L., Andrade J., Staley J.P., Satoh T. (2016). Cancer therapies activate RIG-I-like receptor pathway through endogenous non-coding RNAs. Oncotarget.

[67] Lugade A.A., Sorensen E.W., Gerber S.A., Moran J.P., Frelinger J.G., Lord E.M. (2008). Radiation-Induced IFN-γ Production within the Tumor Microenvironment Influences Antitumor Immunity. J. Immunol..

[68] Kawazu M., Ueno T., Saeki K., Sax N., Togashi Y., Kanaseki T., Chida K., Kishigami F., Sato K., Kojima S. (2022). HLA Class I Analysis Provides Insight Into the Genetic and Epigenetic Background of Immune Evasion in Colorectal Cancer With High Microsatellite Instability. Gastroenterology.

[69] Anderson P., Aptsiauri N., Ruiz-Cabello F., Garrido F. (2021). HLA class I loss in colorectal cancer: Implications for immune escape and immunotherapy. Cell. Mol. Immunol..

[70] thor Straten P., Garrido F. (2016). Targetless T cells in cancer immunotherapy. J. Immunother. Cancer.

[71] Grizzi F., Franceschini B., Biccari S.D., Bossi P., Chiriva-Internati M., Ferrone S. (2013). High frequency of HLA class I antigen processing machinery (APM) component upregulation in primary hepatocellular carcinoma tumors. J. Immunother. Cancer.

[72] Paterson A.C., Sciot R., Kew M.C., Callea F., Dusheiko G.M., Desmet V.J. (1988). HLA expression in human hepatocellular carcinoma. Br. J. Cancer.

[73] Leone P., Shin E.C., Perosa F., Vacca A., Dammacco F., Racanelli V. (2013). MHC Class I Antigen Processing and Presenting Machinery: Organization, Function, and Defects in Tumor Cells. JNCI J. Natl. Cancer Inst..

[74] Wadee A.A., Paterson A., Coplan K.A., Reddy S.G. (1994). HLA expression in hepatocellular carcinoma cell lines. Clin. Exp. Immunol..

[75] Huang J. (2002). HLA class I expression in primary hepatocellular carcinoma. World J. Gastroenterol..

[76] Lei W.Y., Hsiung S.C., Wen S.H., Hsieh C.H., Chen C.L., Wallace C.G., Chang C.C., Liao S.K. (2018). Total HLA Class I Antigen Loss with the Downregulation of Antigen-Processing Machinery Components in Two Newly Established Sarcomatoid Hepatocellular Carcinoma Cell Lines. J. Immunol. Res..

[77] Zhang S., Du K., Gao S., Liu Z., Chen L., Wu X., Li L. (2025). APM-Related gene signature model to predict prognosis and immunotherapy response in hepatocellular carcinoma. Hum. Genet..

[78] Pandha H., Rigg A., John J., Lemoine N. (2007). Loss of expression of antigen-presenting molecules in human pancreatic cancer and pancreatic cancer cell lines. Clin. Exp. Immunol..

[79] Imanishi T., Kamigaki T., Nakamura T., Hayashi S., Yasuda T., Kawasaki K., Takase S., Ajiki T., Kuroda Y. (2006). Correlation between expression of major histocompatibility complex class I and that of antigen presenting machineries in carcinoma cell lines of the pancreas, biliary tract and colon. Kobe J. Med. Sci..

[80] Torres M.J., Ruiz-Cabello F., Skoudy A., Berrozpe G., Jimenez P., Serrano A., Real F.X., Garrido F. (1996). Loss of an HLA haplotype in pancreas cancer tissue and its corresponding tumor derived cell line. Tissue Antigens.

[81] Hiraoka N., Ino Y., Hori S., Yamazaki-Itoh R., Naito C., Shimasaki M., Esaki M., Nara S., Kishi Y., Shimada K. (2020). Expression of classical human leukocyte antigen class I antigens, HLA-E and HLA-G, is adversely prognostic in pancreatic cancer patients. Cancer Sci..

[82] Imai D., Yoshizumi T., Okano S., Uchiyama H., Ikegami T., Harimoto N., Itoh S., Soejima Y., Aishima S., Oda Y. (2017). The prognostic impact of programmed cell death ligand 1 and human leukocyte antigen class I in pancreatic cancer. Cancer Med..

[83] Ryschich E., Nötzel T., Hinz U., Autschbach F., Ferguson J., Simon I., Weitz J., Fröhlich B., Klar E., Büchler M.W. (2005). Control of T-cell-mediated immune response by HLA class I in human pancreatic carcinoma. Clin. Cancer Res..

[84] Cattaneo G., Ventin M., Arya S., Kontos F., Michelakos T., Sekigami Y., Cai L., Villani V., Sabbatino F., Chen F. (2024). Interplay between B7-H3 and HLA class I in the clinical course of pancreatic ductal adenocarcinoma. Cancer Lett..

[85] Zebertavage L.K., Alice A., Crittenden M.R., Gough M.J. (2020). Transcriptional Upregulation of NLRC5 by Radiation Drives STING- and Interferon-Independent MHC-I Expression on Cancer Cells and T Cell Cytotoxicity. Sci. Rep..

[86] Chandrashekar D.S., Karthikeyan S.K., Korla P.K., Patel H., Shovon A.R., Athar M., Netto G.J., Qin Z.S., Kumar S., Manne U. (2022). UALCAN: An update to the integrated cancer data analysis platform. Neoplasia.

[87] Gao J., Shi L.Z., Zhao H., Chen J., Xiong L., He Q., Chen T., Roszik J., Bernatchez C., Woodman S.E. (2016). Loss of IFN-γ Pathway Genes in Tumor Cells as a Mechanism of Resistance to Anti-CTLA-4 Therapy. Cell.

[88] Dhatchinamoorthy K., Colbert J.D., Rock K.L. (2021). Cancer Immune Evasion Through Loss of MHC Class I Antigen Presentation. Front. Immunol..

[89] Lau D., Elliott T. (2025). Imaging antigen processing and presentation in cancer. Immunother. Adv..

[90] Pishesha N., Harmand T.J., Ploegh H.L. (2022). A guide to antigen processing and presentation. Nat. Rev. Immunol..

[91] Gravett A.M., Trautwein N., Stevanović S., Dalgleish A.G., Copier J. (2018). Gemcitabine alters the proteasome composition and immunopeptidome of tumour cells. OncoImmunology.

[92] Pellicciotta I., Yang C.-P.H., Goldberg G.L., Shahabi S. (2011). Epothilone B enhances Class I HLA and HLA-A2 surface molecule expression in ovarian cancer cells. Gynecol. Oncol..

[93] Hu Q., Ye Y., Chan L.-C., Li Y., Liang K., Lin A., Egranov S.D., Zhang Y., Xia W., Gong J. (2019). Oncogenic lncRNA downregulates cancer cell antigen presentation and intrinsic tumor suppression. Nat. Immunol..

[94] Herber D.L., Cao W., Nefedova Y., Novitskiy S.V., Nagaraj S., Tyurin V.A., Corzo A., Cho H.-I., Celis E., Lennox B. (2010). Lipid accumulation and dendritic cell dysfunction in cancer. Nat. Med..

[95] Hu M., Liu D.-A., Wortzel I., Collier P., Nelson T.M., Foox J., Zhong G., Tobias G., Asao T., Bojmar L. (2026). Activated T cell extracellular vesicle DNA transfer enhances antigen presentation and anti-tumor immunity. Cancer Cell.

